# Oxidative stress and regulation of adipogenic differentiation capacity by sirtuins in adipose stem cells derived from female patients of advancing age

**DOI:** 10.1038/s41598-024-70382-x

**Published:** 2024-08-27

**Authors:** Anne Bernhardt, Alan Jamil, Md. Tanvir Morshed, Pia Ponnath, Veronika Gille, Nadine Stephan, Heinrich Sauer, Maria Wartenberg

**Affiliations:** 1 Department of Physiology, Justus Liebig University Giessen, Giessen, Germany; 2 Department of Internal Medicine I, Division of Cardiology, University Hospital Jena, Friedrich Schiller University, Am Klinikum 1, 07747 Jena, Germany

**Keywords:** Adipose stem cells, Oxidative stress, Adipogenesis, Sirtuins, Developmental biology, Stem cells, Molecular medicine, Medical research, Stem-cell research

## Abstract

Patient age is critical for mesenchymal stem cell quality and differentiation capacity. We demonstrate that proliferation and adipogenic capacity of subcutaneous adipose stem cells (ASCs) from female patients declined with advanced age, associated with reduction in cell nucleus size, increase in nuclear lamina protein lamin B1/B2, and lamin A, upregulation of senescence marker p16INK4a and senescence-associated β-galactosidase activity. Adipogenic induction resulted in differentiation of adipocytes and upregulation of adipogenic genes CCAAT enhancer binding protein alpha, fatty acid binding protein 4, lipoprotein lipase, and peroxisome proliferator-activated receptor-γ, which was not affected by the Sirt-1 activator YK-3-237 or the Sirt-1 inhibitor EX-527. Protein expression of the stem cell markers Oct4 and Sox2 was not significantly downregulated with advanced patient age. Mitochondrial reactive oxygen species were increased in ASCs from old-aged patients, whereas protein expression of NADPH oxidases NOX1 and NOX4 was downregulated, and dual oxidase isoforms remained unchanged. Generation of nitric oxide and iNOS expression was downregulated. Protein expression of Sirt-1 and Sirt-3 decreased with patient age, whereas Sirt-2 and Sirt-5 remained unchanged. Induction of adipogenesis stimulated protein expression of Sirt-1 and Sirt-3, which was not affected upon pre-incubation with the Sirt-1-activator YK-3-237 or the Sirt-1-inhibitor EX-527. The Sirt-1 inhibitor Sirtinol downregulated adiponectin protein expression and the number of adipocytes, whereas YK-3-237 exerted stimulatory effects. In summary, our data demonstrate increased oxidative stress in ASCs of aging patients, and decline of adipogenic capacity due to Sirt-1- mediated adiponectin downregulation in elderly patients.

## Introduction

Subdermal fat tissue is a valuable and abundant source of mesenchymal stem cells (MSCs), named ASCs, which may be used in regenerative medicine approaches like wound healing, tissue reconstruction and cell replacement therapies. The differentiation capacities of ASCs comprise mainly the mesodermal cell line, i.e. differentiation into adipogenic, osteogenic, and chondrogenic lineages under appropriate in vitro conditions. Moreover, ASCs were shown to exert immune-modulating, anti-inflammatory and pro-angiogenic properties, presumably through the paracrine release of soluble factors^1^.

Aging is associated with the progressive inability to maintain tissue homeostasis or robustly regenerate tissue after injury or stress. These processes are mediated by tissue-specific stem cells including ASCs, suggesting that impaired stem cell function may underlie central cellular pathophysiologies associated with aging^2^. With increasing life time stem cell numbers are declining and stem cells themselves are aging and may lose their differentiation capacities which limits their use in regenerative medicine^3^. The features of ASC stem cell aging are still under debate. In a previous study proliferation, osteogenic and chondrogenic differentiation potential of human ASCs did not correlate with patient age, whereas a decline in adipogenic differentiation with increasing age was observed^4^. A further study showed a decline in proliferation capacity, differentiation towards osteoblast and adipocytes as well as paracrine action in the elderly patient group^5^.

According to the free radical theory of aging introduced by Denham Harman in 1956^6^ it is proposed that ageing and age-related diseases might be due to the long-term effects of oxidative damage. ROS are physiologically generated within the mitochondrial respiratory chain or by NADPH oxidases which are either expressed in the plasma membrane (e.g. NOX2) of cells or in mitochondria (e.g. NOX1^7^ and NOX4^8^), and are coordinating the intracellular redox milieu. It has been recently discussed that ROS from different intracellular sources can communicate in a process named "ROS-induced ROS release", which is a mechanism for ROS amplification at distinct subcellular compartments^9^. Indeed, it was shown that NOX2 stimulates mitochondrial ROS by activating reverse electron transfer^10^. Whereas excessive ROS, exerting oxidative stress, are upregulated with increasing age, an age-related decrease in the bioavailability of nitric oxide (NO) has been discussed^11^. In a prospective cohort study of 204 subjects it was demonstrated that aging was associated with marked impairment of determinants of NO generation^12^ which may contribute to age-related endothelial cell dysfunction^13^. Oxidative and nitrosative stress are closely regulated by sirtuins (Sirts) which are NAD^+^-dependent enzymes that catalyze the post-translational modification of various enzymes and signaling factors, including deacetylation (Sirt-1-Sirt-7), decrotonylation (Sirt-3), ADP-ribosylation (Sirt-4 and Sirt-6), diacylation (Sirt-6), and desuccinylation, demalonylation, and deglutarylation (Sirt-5)^14^. Above all, Sirt-1 activity has been associated with longevity, which may be due to its role maintaining redox homeostasis by downregulation of ROS formation and upregulation of NO production^15^. In transgenic mice moderately overexpressing Sirt-1 under its own regulatory elements (Sirt-1-tg) aging was associated with lower levels of DNA damage, decreased expression of the ageing-associated gene p16INK4a, a better general health and fewer spontaneous carcinomas and sarcomas, whereas longevity was not affected^16^. In contrast, stimulation of Sirt-1 by the Sirt-1 activator SRT1720 in mice fed a standard diet led to extension in lifespan, delayed onset of age-related metabolic diseases, and improved general health^17^. In senescent cells from human lung fibroblasts (IMR90) and mouse embryonic fibroblasts (MEFs) Sirt-1 protein expression was decreased. This observation was confirmed in tissues of aging mice in which mitotic activity also declines^18^. In a recent study, gene inactivation of Sirt-1 in mouse MSCs caused significant defects and cellular senescence during adipogenic differentiation^19^.

Adiponectin is a hormone released from fat cells into the blood stream and is involved in lipid and glucose metabolism as well as insulin sensitivity. It presumably has a protective role against age-related disease, and thus is an excellent candidate gene for longevity since its expression was both increased in centenarians as well as in their descendants^20^. It was previously shown, that serum adiponectin levels increased with increasing age of healthy subjects and in patients with diabetes, in both men and women^21^. This observation was recently confirmed in a study showing that high-molecular-weight (cHMW) adiponectin levels increased with age until centenarians^22^. A further study showed that adiponectin levels increased linearly with aging in males, whereas it increased dramatically in females until their 50 s^23^. In 3T3-L1 preadipocytes Sirt-1 promoted lipid metabolism, mitochondrial biogenesis and adiponectin expression^24^. On the other hand stimulation of adiponectin receptor 1 (AdipoR1) by adiponectin resulted in activation of AMPK and Sirt-1 by initiating Ca^2+^ influx^25^.

In the present study we investigated the hypotheses that (1) the expression of Sirt-1, Sirt-2, Sirt-3 and Sirt-5 is associated with patient age, (2) correlates with ROS and NO generation, (3) is dependent on the adipogenic capacity and Sirt-1 regulated expression of adiponectin in ASCs derived from subcutaneous fat of female patients of different age groups.

## Materials and methods

Human ASCs were isolated from subdermal fat tissue of female patients during routine surgery and were allocated to different age classes, i.e. 20–34 years (7 patients), 35–49 years (8 patients), 50–64 years (9 patients), 65–80 years (7 patients). Patients used in each age class for experiments were chosen randomly. All cell handling procedures described herein were performed in accordance with the ethical standards laid down in the 1964 Declaration of Helsinki and its later amendments and were approved by the Local Bioethics Committee No. 5092-03/17, AZ202/21. Informed consent was obtained from all subjects and/or their legal guardian(s). The list of patients is shown in supplemental Table 1. Isolation was performed using a modified protocol from a previously published study^26^. Subdermal fat tissue was obtained either by surgery or liposuction. Briefly, fat tissue obtained from surgery was minced, blood vessels and connective tissue were removed, and the tissue was washed several times in phosphate-buffered saline (PBS). Subsequently the tissue and liposuction fluid were enzymatically dissociated by incubation for 1 h with collagenase B (2 mg/ml) at 37 °C and on a shaker. The reaction was stopped with Dulbecco’s Modified Eagle Medium (DMEM) high glucose (Gibco, Life Technologies, Darmstadt, Germany) supplemented with 1.7 mM L-glutamine, 0.84 × non-essential amino acids (NEA) (stock 100 x), 0.84 mM sodium pyruvate, 6 × 10^–3^% 2-mercaptoethanol, 40 U/mL penicillin, and 40 μg/ml streptomycin (all from Sigma-Aldrich,Taufkirchen, Germany) and 12.5% heat-inactivated foetal bovine serum (FBS) (Sigma-Aldrich). Subsequently the sample was centrifuged for 10 min at 447 g to obtain the stromal vascular fraction pellet containing ASCs. For lysis of erythrocytes the cell pellet was incubated for 10 min on ice in PBS containing 160 mM NH_4_Cl. Thereafter the cell suspension was passed through a cell strainer (40 µm), centrifuged for 10 min at 447 g, resuspended in complete DMEM medium and seeded on gelatinized cell culture plates. The stem cell properties of isolated ASCs were checked as previously described by flow cytometry^27^ and were positive for at least 12 passages for CD29, CD44, CD73, CD90, CD105 and CD166. In contrast, no significant expression of the haematopoietic lineage markers CD34 and CD133 and the endothelial cell marker CD31 was observed in any of the earlier and later passages. Cell cultures reaching 90% confluence were enzymatically dissociated using 0.25% trypsin–EDTA solution (Gibco). For long term storage ASCs were cryopreserved and stored in a nitrogen tank at − 196 °C. For the experiments ASCs were passaged for maximum 10 times. For the experiments passaged, adherent ASCs were used.

### Adipogenic differentiation of ASCs

ASCs were cultivated until passage 6. Subsequently cells were enzymatically dissociated and seeded either on T25 and T75 cell culture flasks or on gelatine-coated cover slips in a cell density of 1.5 × 10^4^ cells/cm^2^ and further cultivated in complete DMEM medium containing 3% FBS. When ASCs reached sub-confluence (80–85%) the complete DMEM medium was supplemented with D-Biotin (33 µM), dexamethasone (100 nM), D-Pantothenic hemicalcium salt (17 µM), 3-Isobutyl-1-methylxanthine (IBMX) (500 µM), insulin (100 nM), rosiglitazone (1 µM), 3,3’,5-Triiod-L-thyronine (2 nM), transferrin (10 µg/ml) (all substances from Sigma-Aldrich). Induction treatment was performed for 6 days with one medium change on day 3 of cell culture. After adipogenic induction cell culture medium was exchanged for maintenance medium composed of complete DMEM medium supplemented with D-Biotin (33 µM), dexamethasone (10 nM), D-Pantothenic hemicalcium salt (17 µM), insulin (10 nM), 3,3’,5-Triiod-L-thyronine (2 nM), transferrin (10 µg/ml), and cells were cultivated for further 6 days. Isolation of mRNA and protein was performed on day 12 of adipogenic cell culture.

### Oil Red O staining procedure

To visualize lipid droplets the Oil Red O staining procedure was performed (Li, et al. 2016). Briefly, ASCs grown on cover slips and treated with adipogenic cell culture medium for 12 days were washed twice with phosphate-buffered saline (PBS) and fixed with 1 ml 4% paraformaldehyde (PFA) for 40 min at room temperature, followed by 3 washing steps with PBS. Staining was performed with Oil Red O dissolved in isopropanol (Sigma-Aldrich). Briefly, Oil Red O stock solution was diluted (6:4) in H_2_O and filtered with Whatman filter. Aliquots of 300 µl were added to cells on coverslips and incubated for 1 h on a shaker, at room temperature, in the dark. Subsequently the solution was removed by 4 times washing with H_2_O dest. Lipid droplets were either visualized by transmission light microscopy or by LSM using the fluorescence properties of Oil Red O. Excitation was performed using the 633 nm band of a helium/neon laser. Emission was recorded using a longpass > 670 nm filter.

### Real-time RT-PCR

For the isolation of RNA TRIzol Reagent (Invitrogen) was used according to the manufacturer´s procedures. Total RNA was prepared using Quiashredder columns and a RNeasy mini kit (both Qiagen, Hilden, Germany) according to manufacturer’s recommendations followed by genomic DNA digestion using DNaseI (Invitrogen, Karlsruhe, Germany). cDNA synthesis was performed using 0.5 µg RNA and SuperScript RTase II (Invitrogen). PCR amplification was performed using the primer sets outlined in Table 1. Real-time RT-PCR was performed using the SYBR-Green (Qiagen, Hilden, Germany) and Rotor-Gene Q (Quiagen). Relative expression values were obtained by normalizing CT values of the tested gene in comparison with CT values of the housekeeping gene using the 2^(-ΔΔCT)^-method. Glucuronidase ß (GUSB) was used as control in the comparative C_T_ method.

**Table 1:** RT-PCR primer sequences.

Target gene	Sequence
*CEBPA*	*Forward: 5 ‘-*CCTTGTGCCTTGGAAATGCAAAC *Reverse: 5 ‘-*CTGCTCCCCTCCTTCTCTCA
*FABP4*	*Forward: 5 ‘-*TCAGTGTGAATGGGGATGTGAT *Reverse: 5 ‘-*TCTGCACATGTACCAGGACACC
*GUSB*	*Forward:* 5 ‘-AAACGATTGCAGGGTTTCAC *Reverse:* 5 ‘-CTCTCGTCGGTGACTGTTCA
*LPL*	*Forward:* 5 ‘-CAGGATGTGGCCCGGTTTAT *Reverse:* 5 ‘-GGGACCCTCTGGTGAATGTG
*NANOG*	*Forward:* 5 ‘-GATTTGTGGGCCTGAAGAAA *Reverse:* 5 ‘-AAGTGGGTTGTTTGCCTTTG
*OCT-3/4*	*Forward:* 5 ‘-CTGAGGGCGAAGCAGGAG *Reverse:* 5 ‘-AATAGAACCCCAGGGTGAG
*PPARγ*	*Forward:* 5 ‘-CTATTGACCCAGAAAGCGAT *Reverse:* 5 ‘-CGTAATGTGGAGTAGAAATGC
*SOX2*	*Forward*: 5 ‘-ACACCAATCCCATCCACACT *Reverse:* 5 ‘-GCAAACTTCCTGCAAAGCTC

### Western blot analysis

Western blot assays were carried out after detaching ASCs with 0.25% Trypsin/EDTA from culture flasks, washing in PBS and lysing the cells twice for 10 min on ice after ultra sound treatment (10 s, 30–40% power; ultra sound homogeniser Sonopuls UW 70, BANDELIN Electronic, Berlin, Germany) in RIPA lysis buffer (50 mM Tris–HCl (pH 7.5), 150 mM NaCl, 1 mM EDTA (pH 8.0), 1 mM glycerophosphate, 0.1% SDS, 1% Nonidet P-40) supplemented with protease inhibitor cocktail (Biovision, Hannover, Germany) and phosphatase inhibitor cocktail (Sigma-Aldrich). Samples were centrifuged at 13,000 g for 10 min at 4 °C to pellet the debris and to collect the supernatant. After determination of the protein concentration using the Pierce BCA Protein Assay Kit (Thermo Fisher Scientific, Waltham, MA, USA), 20 µg of whole protein samples were heated for 10 min at 70 °C, separated in NUPAGE 4–12% Bis–Tris gradient mini gels (150 V for 90 min) and transferred to PVDF membranes by the XCell SureLock Mini-Cell Blot Module (Invitrogen) at 180 mA for 90 min. Membranes were blocked with 5% (wt/vol) dry fat-free milk powder in Tris-buffered saline with 0.1% Tween (TBS-T) for 60 min at room temperature. Incubation with primary antibody was performed at 4 °C overnight. Used primary antibodies were: rabbit anti NOX1 (dilution 1:500) (Proteintech, Planegg-Martinsried, Germany, cat.no. 17772-1-AP), rabbit anti NOX2 (dilution 1:1000) (Proteintech, cat.no. 19013-1-AP), rabbit anti NOX4 (dilution 1:1000) (Abcam, Cambridge, UK, cat.no. ab109225), rabbit anti DUOX1 (dilution 1:1000) (Bioss, Woburn, MA, USA, cat.no. bs-11431R), rabbit anti DUOX2 (dilution 1:1000) (BioTechne, Wiesbaden, Germany, cat.no. NB110-64576), rabbit anti iNOS (dilution 1:1000) (Thermo Fisher Scientific, cat.no. PA1-036), mouse anti Lamin B1 + B2 (dilution 1:1000) (Abcam, cat.no. ab4825), mouse anti Lamin A/C (dilution 1:1000) (Cell Signaling, cat.no. 4777S), mouse anti Sirt-1 (dilution 1:2000) (Bio-Techne, cat.no. NBP1-51641), mouse anti Sirt-2 (dilution 1:10000) (Proteintech, cat. no. 66410-1-lg), rabbit anti Sirt-3 (dilution 1:1000) (Biorbyt, Cambridge, UK, cat.no. orb247889), rabbit anti Sirt-5 (dilution 1:750) (Proteintech, cat.no. 15122–1-AP), rabbit anti Oct 4 (dilution 1:500) (BioTechne, cat. no. NB100-2379), rabbit anti Sox2 (dilution 1:2000) (Thermo Fisher, cat. no. PA1-16968), goat anti Nanog (dilution 1:10000) (Abcam, cat. no. ab 177095), rabbit anti PPARγ (dilution 1: 500) (Abcam, cat.no. ab209150), rabbit anti adiponectin (dilution 1:2000) (Thermo Fisher Scientific, cat. no. 701148), mouse anti Vinculin (dilution 1:1000) (Sigma-Aldrich, cat.no. V9131), rabbit anti Vinculin (dilution 1:1000) (Abcam, cat.no. ab129002), rabbit anti GAPDH (dilution 1:2000) (Abcam, cat. no. ab9485). After washing three times with 0.1% TBS-T for 10 min, the membranes were incubated with a corresponding horseradish peroxidase (HRP)-conjugated secondary antibody (dilution 1:1000 (1:5000 for PPARγ) (Abcam, cat.no. ab205722, ab205724) for 60 min at room temperature. After the washing process with 0.1% TBS-T, the blot was developed using ECL to produce a chemiluminescence signal. For quantification, the density of protein bands on the western blot image, which was acquired using the peqlab gel documentation system (VWR, Darmstadt, Germany), was assessed by Image J. For each sample, the ratio of each target protein to the respective housekeeping protein was calculated. The final quantification reflects the relative amounts of protein compared to the youngest age group (20–34 y). Information on all western blots performed in this study is presented in supplementary files.

### Immunohistochemistry

As primary antibodies mouse anti Sirt-1 (dilution 1:100) (Bio-Techne, cat.no. NBP1-51641), mouse anti Sirt-2 (dilution 1:100) (Proteintech, cat. no. 66410-1-lg), rabbit anti Sirt-3 (dilution 1:100) (Biorbyt, Cambridge, UK, cat.no. orb247889), rabbit anti Sirt-5 (dilution 1:100) (Proteintech, cat.no. 15122-1-AP), rabbit anti p16INK4a (dilution 1:100) (Invitrogen, cat.no. PA5-20379) were used. ASCs cultivated on coverslips were fixed in 4% PFA on ice for 20 min, washed 1–2 times with phosphate-buffered saline (PBS) supplemented with 0.01% Triton-X-100 (Sigma-Aldrich) (0.01% PBST) and permeabilized for 10 min with 1% PBST. Blocking against unspecific binding was performed for 60 min with 10% fat-free milk powder dissolved in 0.01% PBST (blocking solution). The cells were subsequently incubated overnight with primary antibody dissolved in blocking solution. Cells were thereafter washed three times with 0.01% PBST and re-incubated for 1 h at room temperature in dark with either a Cy2 goat anti rabbit, Cy2 goat anti mouse antibody (dilution 1:100) in blocking solution. After washing three times in 0.01% PBST the cells were stored in PBS until inspection. For staining of cell nuclei the nuclear stain DRAQ5 (Thermo Fisher Scientific) was used (dilution 1:2000) and excited at 633 nm. Fluorescence recordings were performed by means of a confocal laser scanning (LSM) setup (Leica TCS SP2, Wetzlar, Germany). The confocal setup was equipped with a 5 mW helium/neon laser, single excitation 633 nm, a 0.5 mW helium/neon laser, single excitation 543 nm, and an argon ion laser, single excitation 488 nm.

### Nucleus area measurement

To determine the nucleus area, ASCs were plated in 24 well plates (50,000 cells per well), led adhered overnight, fixed (4% PFA, 20 min, room temperature), permeabilized in 1% PBST and stained with the nucleus-specific fluorescence dye DRAQ5 (1:2000 in PBS; BioLegend) for 30 min at room temperature. Microscopic pictures were recorded by LSM (excitation 633 nm, emission 680–720 nm). The nucleus area was measured using the image analysis software ImageJ.

### Measurement of NO generation

NO generation was evaluated by the use of the cell permeable specific fluorescent NO indicator 4,5-Diaminofluoresceindiacetat **(**DAF-2DA) (Sigma, cat.no 251505). After incorporation into cells DAF-2 reacts rapidly with NO in the presence of oxygen to yield the highly fluorescent compound triazolofluorescein (DAF-2 T). Cells grown on cover slips to confluency were incubated for 30 min with 10 µM DAF-2DA dissolved in E1 buffer, containing (in mM) NaCl 135, KCl 5.4, CaCl_2_ 1.8, MgCl_2_ 1, glucose 10, HEPES 10 (pH 7.4 at 23 °C). Subsequently, coverslips were transferred to an incubation chamber mounted to the inspection table of the confocal setup, and DAF-2 T fluorescence was recorded in single cells using the 488 nm band of the argon-ion laser of the confocal setup. Emission was recorded at > LP 515 nm.

### Measurement of cytoplasmic and mitochondrial ROS

For the determination of cytoplasmic ROS the fluorescence indicator 2',7'-Dichlorodihydrofluorescein-diacetate (H_2_DCF-DA) (Thermo Fisher, cat. no. D399) was used. Mitochondrial ROS were determined by MitoSOX red indicator (Thermo Fisher, cat. no. M36008). ASCs on coverslips were incubated in E1 buffer containing (in mM): NaCl 135, KCl 5.4, CaCl2 1.8, MgCl2 1, glucose 10, HEPES 10 (pH 7.4 at 23 °C), buffer supplemented with 10 µM H_2_DCF-DA (H_2_DCF-DA stock solution, 10 mM, dissolved in DMSO). Incubation was performed for 20 min in a CO_2_ incubator (37 °C). For fluorescence excitation of DCF, the 488 nm band of the argon ion laser of the LSM was used. Fluorescence emission was recorded at an emission band of 515–550 nm. MitoSOX red (5 µM) was added to DMEM cell culture medium without additives (MitoSOX stock solution, 5 mM, dissolved in DMSO) and incubated for 10 min at 37 °C in the CO_2_ incubator. MitoSox Red was excited at 514 nm. Fluorescence emission was recorded using a band pass filter 550–600 nm.

### Senescence-associated β-galactosidase fluorescence assay

Activity of senescence-associated β-galactosidase was determined by use of the CellEvent Senescence Green Detection Kit (Thermo Fisher Scientific). Senescence Green Probe is a fluorescent-based reagent that contains two galactoside moieties, making it specific to β-galactosidase. The enzyme-cleaved product is trapped within the cell due to covalent binding of intracellular proteins and emits a fluorogenic signal that has excitation/emission maxima of 490/514 nm. Briefly, PFA-fixed (4% PFA, 10 min) confluent ASC cell cultures on coverslips were incubated for 2 h at 37 °C under light protection with Senescence Green Probe (dilution 1:1000) followed by washing in PBS. Cellular fluorescence was recorded using LSM (excitation 488 nm, emission 500–555 nm), and fluorescence images were analyzed by computer-assisted image analysis using the Fiji software package.

### Statistical analysis

Data are given as mean values ± SD, with *n* denoting the number of experiments unless otherwise indicated. One-way ANOVA followed by Tukey's post hoc test was used to compare the differences between continuous data (> 3 groups), while differences between 2 groups were compared using an unpaired Mann Whitney U test.

### Informed consent

The corresponding author confirms, that informed consent was obtained from all authors of the present study

## Results

### Correlation of ASC proliferation, size of cell nuclei and expression of lamins with increasing patient age

**Figure 1:**
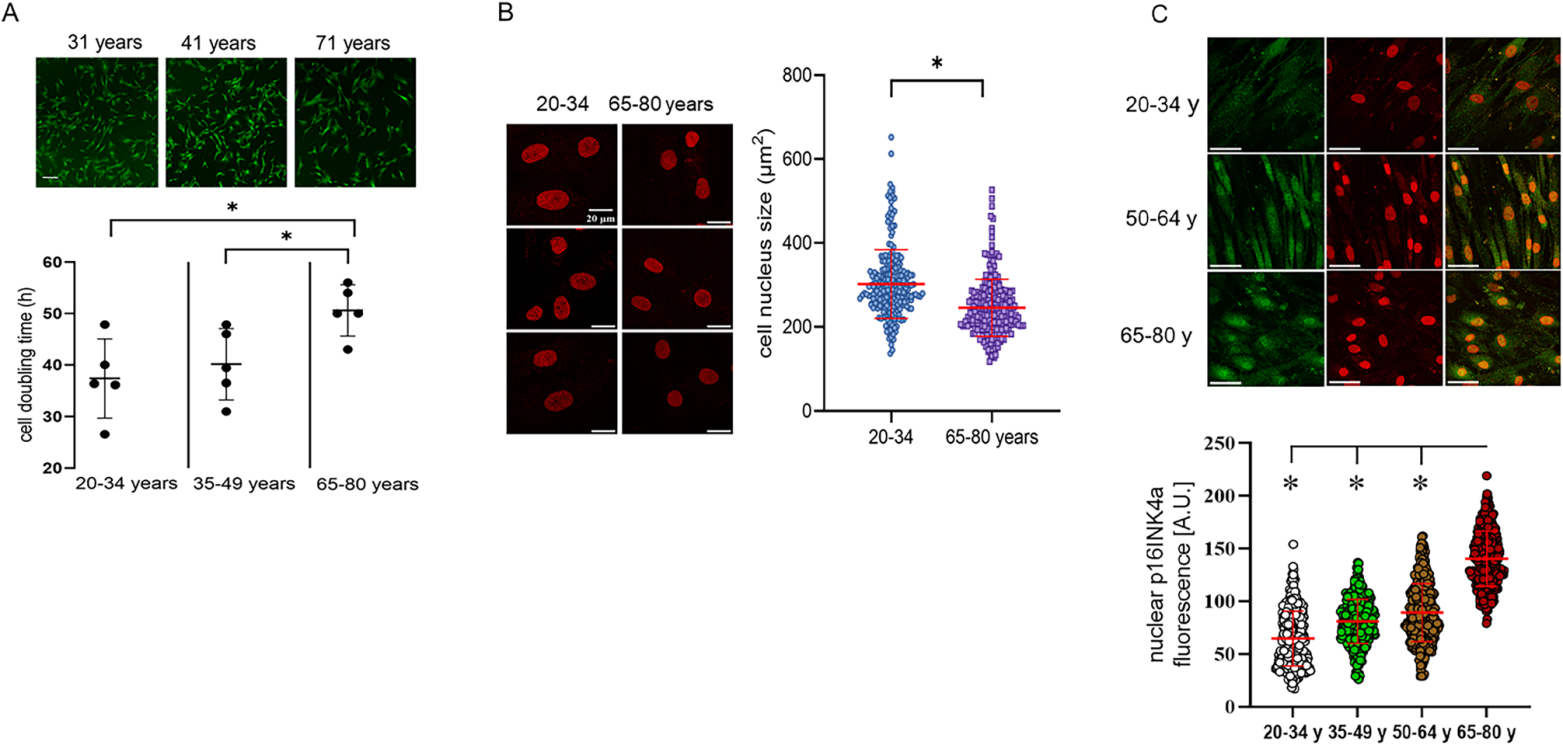
Cell doubling times, size of cell nuclei and p16INK4a expression in ASC from patients of increasing age. **A** Assessment of cell proliferation in in young (20–34 years), middle aged (35–49 years) and old aged (65–80 years) female patients. ASCs (10^4^ cells/ml) were seeded onto coverslips and cells positive for the cell vitality marker Calcein, AM were assessed every 24 h for 96 h by microscopic inspection (n = 5). The bar represents 200 µm. *p < 0.05, statistically significant as indicated. **B** Size of cell nuclei in ASCs of 20–34 and 65–80 year-old patients. A number of 3 individual patients was investigated in each age group, and each experiment was repeated 3 times (n = 3). Cell nuclei were stained with DRAQ5 and the size of cell nuclei was assessed by computer-assisted image analysis. The bar represents 20 µm. *p < 0.05, statistically significant as compared to the 20–34 year-old group. **C** Nuclear expression of the senescence marker p16INK4 in ASCs of female patients of increasing age. Upper panel: representative immunohistochemical stainings of p16INK4a (green), nuclear DRAQ5 (red) and overlay in 20–34, 50–64, and 65–80 year-old patients. The bar represents 50 µm. Lower panel: Scatter blot (means ± SD) of single cell nuclear p16INK4a fluorescence in 20–34 (n = 3 patients), 35–49 (n = 3 patients), 50–64 (n = 3 patients) and 65–80 (n = 3 patients) year-old patients. Each experiment was repeated 3 times. *p < 0.05, statistically significant as compared to the 65–80 year-old group.

**Figure 2:**
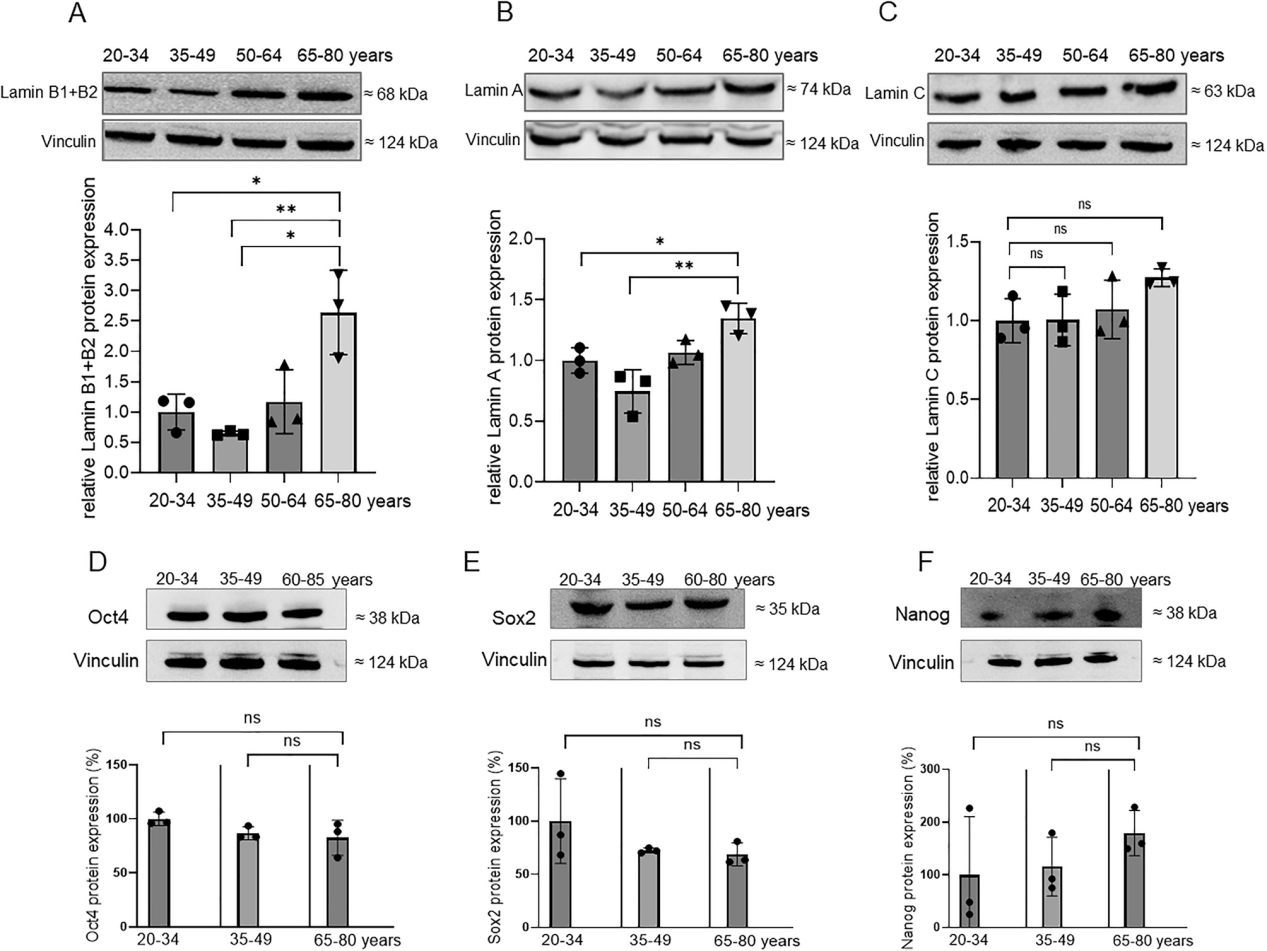
Protein expression of lamins and stem cell markers in relation to patient age. **A** lamin B1/B2, **B** lamin A, **C** lamin C, **D** Oct4, **E** Sox2, **F** Nanog. Shown are representative western blots. The bar charts show the means ± SD of n = 3 patients in each age group. Each experiment was repeated 3 times. *p < 0.05, **p < 0.01, statistically significant as indicated.

MSC quality and stem cell numbers may decrease in elderly patients as compared to young patients. This may be associated with downregulation of the proliferation rate. To assess this issue, cell doubling times of ASCs grown in cell culture on glass cover slips were investigated in young (20–34 years), middle aged (35–49 years) and old aged (65–80 years) female patients. Our data demonstrated that the cell doubling time significantly increased from 37.4 ± 7 h to 40.1 ± 6 and 51 ± 4 h in young, middle-aged and old-aged patients, respectively, supporting the notion that cell division is slowed-down in the elderly patient cohort (Fig. 1A). Notably, the decline of proliferation rate was associated with decrease in the size of cell nuclei in old-aged patients (Fig. 1B). The decline in proliferation capacity may be associated with increased cell senescence. We therefore assessed nuclear expression of the senescence marker p16INK4a in different ASC age classes (Fig. 1C), and performed a fluorescence-based enzymatic assay for senescence-associated β-galactosidase activity (suppl. Fig. 1). Our data demonstrated significant upregulation of nuclear p16INK4a fluorescence in the old-aged patient group and a non-significant upregulation of senescence-associated β-galactosidase activity. Since the nuclear lamina network is formed by lamins, belonging to the family of type V intermediate filaments, expression of lamin B1/B2 and lamins A and C were investigated in patients during the aging process. It was shown that lamin B1/B2 (Fig. 2A) and lamin A (Fig. 2B) protein expression was significantly upregulated with increasing patient age, and a significant linear trend of upregulation was evident for lamin C (Fig. 2C). These results of increased expression of lamins in elderly patients support the notion of augmented nuclear stiffness in aged tissues, which may reduce the ability to rearrange under mechanical stress.

### Protein expression of stem cell genes in ASCs during patient aging

Plasticity of ASCs may be correlated with the protein expression level of stem cell genes. We therefore investigated the protein expression level of Oct4, Sox2 and Nanog, which are the core factors in regulation of stem cell pluripotency. It was shown that Oct4 (Fig. 2D) and Sox2 (Fig. 2E) protein levels gradually, but not significantly, decreased with increasing patient age, whereas Nanog was non-significantly upregulated (Fig. 2F). No obvious age-related changes in mRNA expression of stemness genes were observed using real time RT-PCR (data not shown).

### Age-dependent protein expression of Sirt-1, Sirt-2, Sirt-3, and Sirt-5 in ASCs

**Figure 3:**
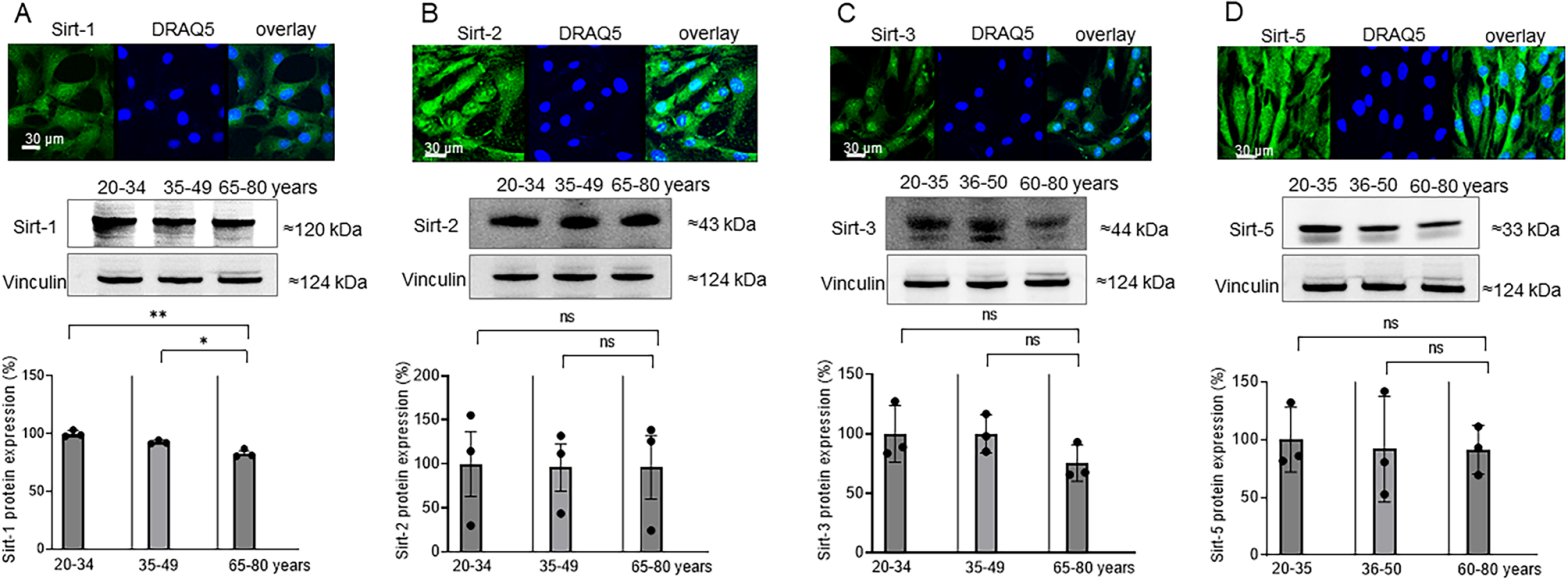
Protein expression of sirtuins in relation to patient age. **A** Sirt-1, **B** Sirt-2, **C** Sirt-3, **D** Sirt-5. Upper row, representative immunostainings for sirtuins (green) in ASCs obtained from 35 to 49 year-old female patients. Cell nuclei were counterstained with DRAQ5 (blue). Overlay images are presented in green/blue color. The bars represent 30 µm. Middle row, representative western blots of Sirt-1, Sirt-2, Sirt-3 and Sirt-5 expression in age classes 20–34 years, 35–49 years, 65–80 years, Vinculin was used as loading control. Lower row, bar charts representing the means ± SD of experiments derived from 3 patients in each age group. Each experiment was repeated 3 times.*p < 0.05, **p < 0.01, statistically significant as indicated.

Since sirtuins have been associated with longevity by—among others—preventing DNA damage, strengthening the antioxidative defense and controlling inflammatory reactions^28^. We therefore investigated Sirt-1, Sirt-2, Sirt-3, and Sirt-5 protein expression in young (20–34 years), middle aged (35–49 years) and old aged (65–80 years) female patients (Fig. 3A–D). Immunohistochemical analysis showed that all investigated sirtuins were expressed in ASCs. Western blot analysis demonstrated that Sirt-1 expression significantly and Sirt-3 non-significantly decreased with patient age. No age dependency was observed for Sirt-2 and Sirt-5.

### ROS and NO generation in ASCs of patients during aging

**Figure 4:**
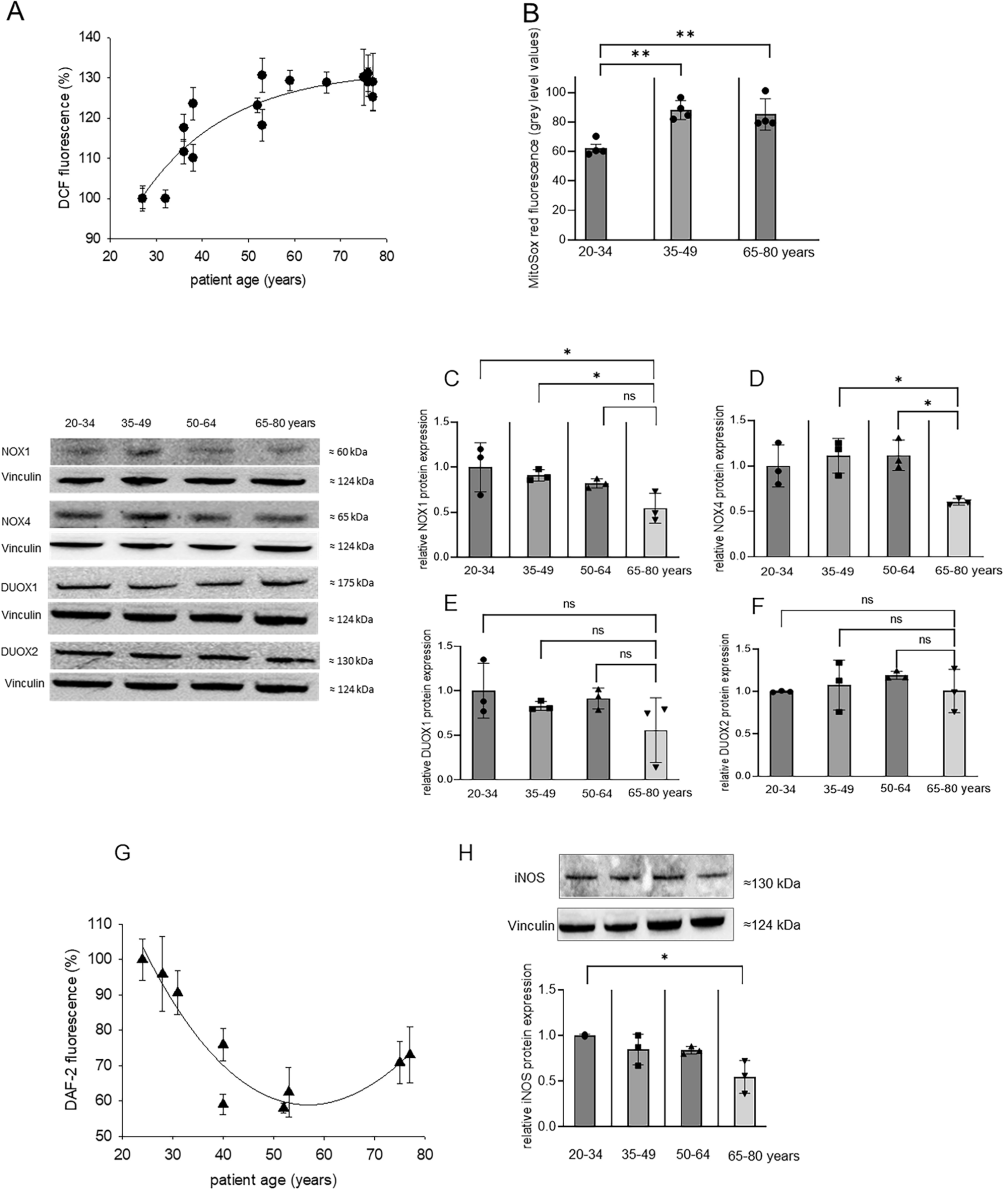
Cytoplasmic and mitochondrial ROS generation, protein expression of ROS-generating enzymes, generation of NO and expression of iNOS in relation to patient age. **A** ROS were assessed in ASCs of individual patients using the fluorescent ROS indicator H_2_DCF-DA. Shown are the means ± SD of n = 3 experiments for each patient. Data are presented as relative DCF fluorescence (%) in relation to the youngest investigated patient (27 year-old). **B** MitoSox red fluorescence in the age groups 20–34, 35–49 and 65–80 years. Data are presented as grey level values obtained from single cells. The bar charts represent the means ± SD of 1 patient in each age group which was repeated 4 times. *p < 0.05, **p < 0.001, statistically significant as indicated. Western blot experiments were performed for **C** NOX1, **D** NOX4, **E** DUOX1, **F** DUOX2. Left, representative western blots in the age groups 20–34, 35–49, 50–64, 65–80 years. Vinculin was used as loading control. Right, bar charts representing the means ± SD of 3 patients in each patient group. *p < 0.05, statistically significant as indicated. **G** NO was assessed in ASCs of individual patients using the fluorescent NO indicator DAF-2DA. Shown are the means ± SD of n = 4 experiments for each patient. Data are presented as relative DAF-2 fluorescence (%) in relation to the youngest investigated patient (24 year-old). **H** Expression of iNOS protein expression in relation to patient age. Above, representative western blot in the age groups 20–34, 35–49, 50–64, 65–80 years. Vinculin was used as loading control. Below, bar charts representing the means ± SD of 3 patients in each patient group. *p < 0.05, statistically significant as indicated.

Sirtuins have been discussed to control oxidative stress and stimulating NO production. NO exerts protective effects on aging cells^29^. We therefore assessed ROS and NO generation in ASCs of patients by using the cytoplasmic ROS-specific fluorescence dye H_2_DCF-DA, the mitochondria-specific ROS indicator MitoSOX Red, and the NO-sensitive dye DAF-2DA. Our data demonstrated that ROS generation significantly increased with patient age (Fig. 4A). MitoSOX Red data identified mitochondria as source of age-dependent ROS generation (Fig. 4B). The increase in mitochondrial ROS generation was counter-balanced by downregulation of NOX1 and NOX4 protein expression with increasing patient age, whereas NOX2 expression was undetectable (Fig. 4C,D). Protein expression of the ROS-generating enzymes DUOX1 and DUOX2 remained unchanged during patient aging (Fig. 4E,F). In contrast to the observed upregulation of ROS, downregulation of NO production was observed with advanced patient age (Fig. 4G) which may indicate loss of oxidative stress protection during aging. Consequently, protein expression of iNOS was decreased in elderly patients (Fig. 4H).

### Age-dependent adipogenesis of ASCs and correlation with sirtuin expression

**Figure 5:**
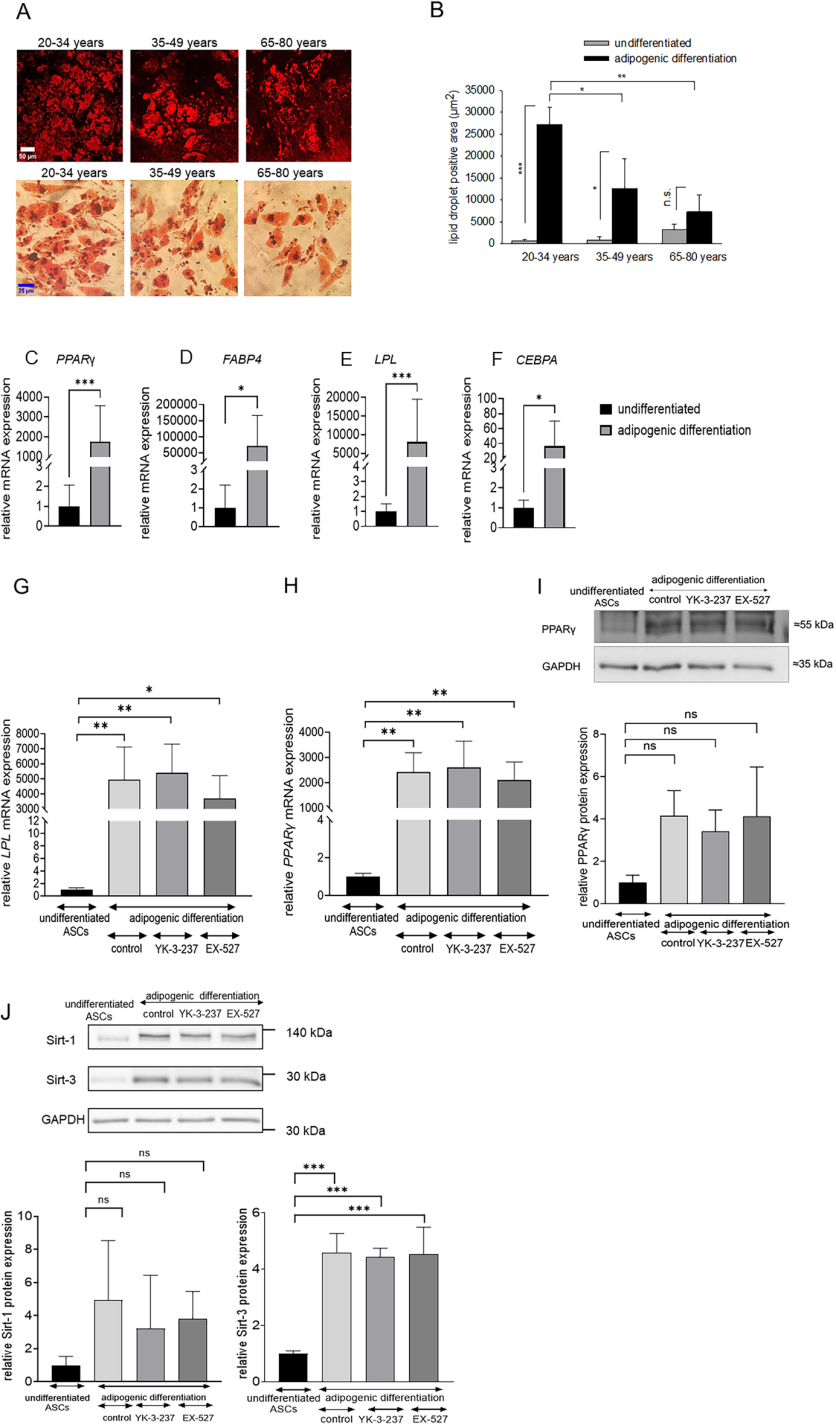
Differentiation of Oil Red O-positive adipocytes in relation to patient age. Effect of the sirtuin activator YK-3-237 and the sirtuin inhibitor EX-527 on mRNA expression of the adipogenic genes LPL and PPARγ, and on protein expression of PPARγ, Sirt-1 and Sirt-3. **A**, **B** ASCs (20–34, 35–49, 65–80 years) remained untreated (control) or were subjected to a adipogenic differentiation protocol. After 12 days of adipogenic induction cells were stained with the adipocyte labeling solution Oil Red O and either fluorescence was recorded by LSM or cells were visualized by bright field light microscopy. The lipid droplet-positive cell area was determined in 512 × 512 pixel images by the image analysis software of the LSM. **A** Representative Oil Red O-stained cell cultures as evaluated by LSM (upper row, bar 50 µm) and bright field light microscopy (lower row, bar 25 µm). **B** Bar chart showing means ± SD of the lipid droplet-positive cell area (µm^2^) of 3 individual patients (n = 3 for each data point). *p < 0.05, statistically significant as indicated. **C**–**F** RT-PCR analysis of mRNA expression for the adipogenesis-associated genes *PPARγ* (**C**), *FABP4* (**D**), *LPL* (**E**), *CEBPA* (**F**). GUSB was used as house-keeping gene. Subjects were randomly selected from the 35–49 year-old group (n = 3). *p < 0.05, **p < 0.01, ***p < 0.001, statistically significant as indicated. (G-J) ASCs derived from 35 to 49 year-old patients remained untreated (control) or were subjected for 6 days to adipogenic induction procedure. After further 6 days of cell culture mRNA and protein was isolated. During the whole adipogenic procedure YK-3-237 (1 µM) and EX-527 (1 µM) were present in the incubation medium. GUSB and GAPDH were used as house-keeping gene and loading control, respectively. **G** mRNA expression of *LPL*. **H** mRNA expression of PPARγ. **I** Protein expression of PPARγ. **J** Protein expression of Sirt-1 and Sirt-3. In western blot experiments representative experiments are shown. The bar charts in western blot experiments show the means ± SD of n = 3 experiments derived from individual patients. *p < 0.05, **p < 0.01, ***p < 0.001, statistically significant as indicated.

The capacitiy of adipogenic differentiation of ASCs may be affected by the age of patients. To investigate this issue, undifferentiated ASCs were subjected for 12 days to adipogenic differentiation medium. Adipogenic induction resulted in differentiation of Oil Red O-positive adipocytes. The adipogenic capacity declined with advanced patient age as indicated by the Oil Red O-positive cell area in differentiated ASC cell cultures (Fig. 5A,B). To correlate adipogenesis to sirtuin activity we investigated mRNA expression of adipogenesis-associated genes. Adipogenic induction resulted in mRNA upregulation of the adipogenic genes *PPAR-γ* (Fig. 5C), *FABP4* (Fig. 5D), *LPL* (Fig. 5E), and *CEBPA* (Fig. 5F). The mRNA expression of *LPL* and *PPAR-γ* was not affected upon pre-treatment with either the Sirt-1 activator YK-3-237 (1 µM) or the Sirt-1 inhibitor EX-527 (1 µM) (Fig. 5G,H). Likewise, adipogenesis-induced upregulation of PPAR-γ protein expression was not affected upon either Sirt-1 activation or inhibition (Fig. 5I). Moreover, adipogenic induction did not significantly change mRNA expression of Sirt-1, Sirt-2, Sirt-3 and Sirt-5 (data not shown). Interestingly, adipogenic induction increased protein expression of Sirt-1 and Sirt-3, which was not affected upon pre-incubation with YK-3-237 or EX-527 (Fig. 5J).

### Downregulation of adiponectin expression and adipogenesis upon Sirt-1 inhibition

**Figure 6:**
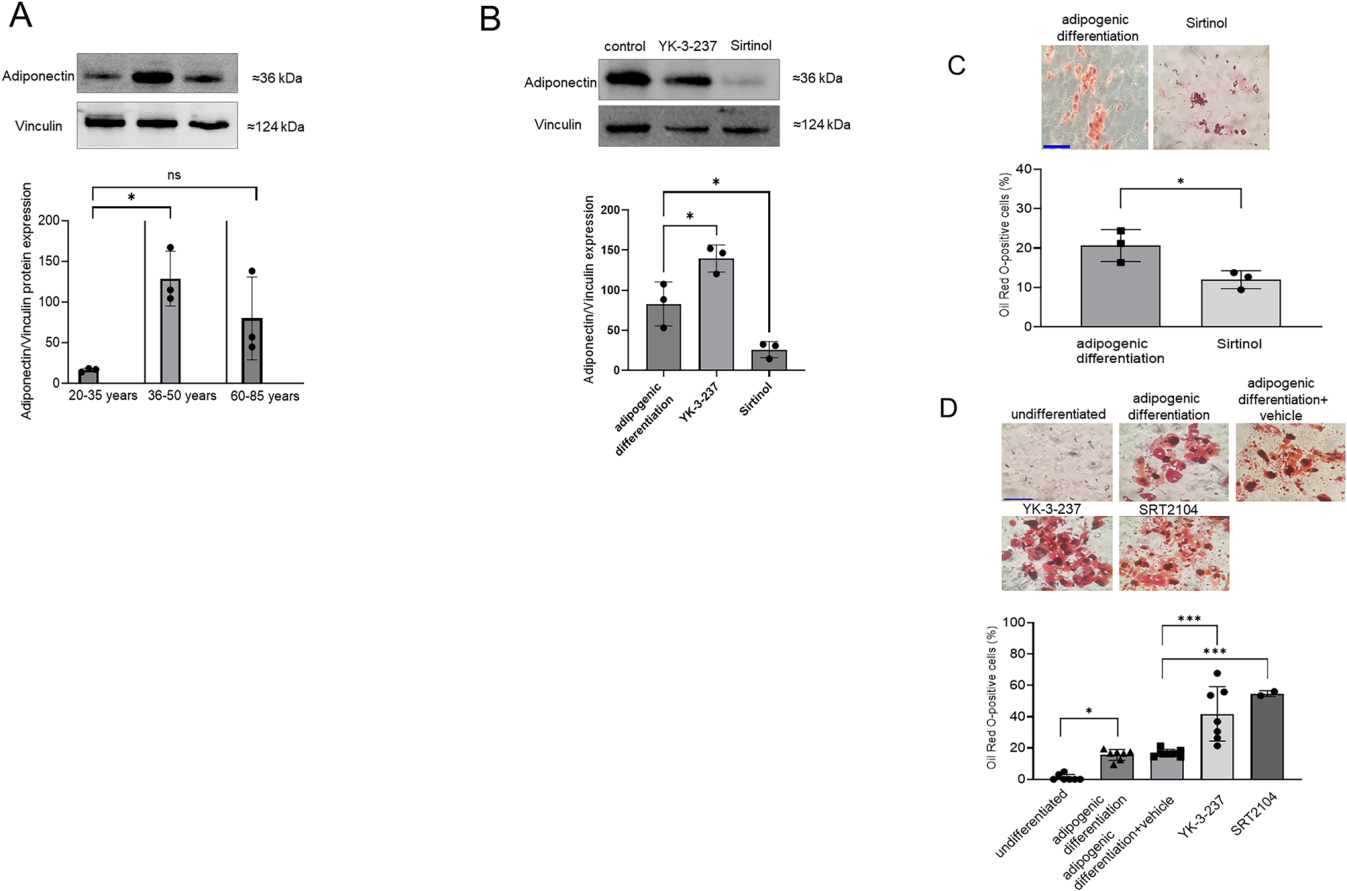
Protein expression of adiponectin in relation to patient age and effect of the Sirt-1 activator YK-3-237 and the Sirt-1 inhibitor Sirtinol. **A** Adiponectin protein expression in 20–34, 35–49 and 65–80 year-old patients following 12 days of adipogenic differentiation. Shown is a representative western blot, Vinculin was used as loading control. The bar chart shows the means ± SD of n = 3 experiments obtained from individual patients. **B** Effect of YK-3-237 and Sirtinol on adiponectin protein expression. Shown is a representative western blot. The bar chart represents the means ± SD from 3 patients of the 65–80 year-old patient group (n = 3) for each data point. **C**, **D** Effect of the Sirt-1 inhibitor Sirtinol (3 patients) (**C**), and the Sirt-1 activators YK-3-237 (3 patients) and SRT2104 (2 patients) (**D**) on the differentiation of Oil Red O-positive adipocytes in patients of the 65–80 year-old group. The agents were present during the whole time course of adipogenic differentiation. The bar represents 50 µm. *p < 0.05, ***p < 0.001, statistically significant as indicated.

Since the adipocyte hormone adiponectin may promote adipogenesis^30^ we investigated adiponectin protein expression in undifferentiated ASCs and upon chemically-induced adipogenesis. In undifferentiated ASCs adiponectin expression was absent (data not shown), whereas treatment with adipogenic cocktail induced adiponectin expression with the highest expression in the 35–49 year-old patient group (Fig. 6A). Notably adiponectin expression was significantly downregulated by the Sirt-1 inhibitor Sirtinol (60 µM) in the old aged (65–80 years) patient group, whereas the Sirt-1 activator YK-3-237 (5 µM) exerted stimulatory effects (Fig. 6B). Moreover, treatment with Sirtinol significantly inhibited the differentiation of Oil Red O-positive adipocytes (Fig. 6C), whereas the Sirt-1 activators YK-3-237 and SRT2104 increased the percentage of Oil Red O-positive adipocytes (Fig. 6D). These data underscore the notion that adipocyte differentiation from ASCs is regulated by an adiponectin-mediated, Sirt-1-dependent, mechanism.

## Discussion

In the current study, stem cell properties of ASCs derived from subcutaneous fat tissue of female patients of increasing age were investigated. We chose the subcutaneous fat since subcutaneous and visceral fat depots differently contribute to obesity and its metabolic comorbidities^31^. Male individuals were excluded to avoid gender bias since the fat tissue mass distribution is different between males and females. Women have more subcutaneous adipose tissue, creating a “pear shape” appearance, while men have fat, predominantly distributed to the visceral adipose tissue around the abdominal organs creating an “apple shape” body habitus^32^. Moreover, it has been stated that regulation of secretion of a number of products from adipose tissue are under the control of sex steroids, which act through a wide variety of mechanisms to tailor metabolism to the unique needs of each sex^33^.

Our data demonstrate that the proliferation capacity of ASCs declined with increasing patient age, thus confirming previous studies on reduced cell doubling times of ASCs in elderly patients^5,34^. Several studies performed on somatic cells derived from laboratory animals and humans have shown that the replication rate of cells is decreasing with advanced donor age^35^, which is associated with increased activity of senescence-associated ß-galacosidase^36^. Indeed the data of the present study showed a moderate stimulation of ß-galacosidase activity and significantly increase in expression of the senescence marker p16INK4a which decelerates cell division by delaying cell cycle transition from the G1 phase to the S phase. p16INK4a has been established as reliable senescence marker and was demonstrated to limit stem cell plasticity^37^. In the present study cell cycle-active cells with a passage number of less than 10 passages were used, which is much less than the Hayflick limit of replicative senescence after approximately 50 cell divisions^38^.

Interestingly the data of the present study demonstrated a reduced size of cell nuclei in the old-aged patient group. Moreover, no obvious wrinkling nuclear membrane irregularities of cell nuclei were evident in elderly patients. Nuclear shape anomalies have been associated with laminopathies like the Hutchinson Gilford progeria syndrome (HGPS), which is genetically caused by mutations in the Lamin A/C gene (*LMNA*) and results in accelerated ageing of affected patients^39^. In HGPS patients decreased nuclear volumes and increased nuclear stiffness was observed which was associated with decreased motility and migration capacity of cells^40^. Previous studies have shown that irradiation-induced senescence resulted in increased sizes of cell nuclei^41–43^. However, nuclear enlargement is also a feature of malignant cells with high proliferation rate and active cell metabolism^44,45^. The data of the present study showed increased expression of lamin B1/B2 and lamin A and lamin C in ASCs of elderly patients. The association of lamin expression levels to cellular senescence is still conflicting. Previous studies have shown that senescence is associated with decreased lamin B1^46^, whereas others evidenced that oxidative stress-induced senescence was associated with increased lamin B1 accumulation^47^. Interestingly, an increase in senescence-associated ß-galactosidase (SA-β-gal)–positive cells was observed, when lamin B1-depleted cells were grown under sparse conditions. This led to the conclusion that lamin B1 depletion does not directly cause senescence unless it is accompanied by an additional stressor, and implicated that lamin B1 loss is a hallmark and consequence but not a cause of senescence^48^.

Sirts have been previously associated with longevity and play a crucial role in the antioxidant defense^49^. It was shown that Sirt-1 was significantly and Sirt-3 tendentially downregulated in the cohort of elderly patients. Changes in Sirt expression were paralleled by upregulation of ROS generation presumably via the mitochondrial respiratory chain. Coenzyme Q_10_ (CoQ_10_) is a central and rate-limiting compound of the mitochondrial respiratory chain and is central to the redox balance of the cell^50^. During aging CoQ_10_ deficiency occurs which may contribute to increased oxidative stress^51^. The mitochondrial NAD^+^/NADH ratio consequent to CoQ_10_ deficit can be expected to slow down the activity of Sirt-1 and Sirt-3 by decreasing availability of their substrate NAD^+^^52^. Moreover, CoQ_10_ deficiency was discussed to decrease the stability of Sirt-1 protein ^52^. The observed decline in NOX1, NOX4 and DUOX1 expression with increasing patient age may be a compensatory cellular reaction to cope with age-related oxidative stress and downregulation of Sirt expression. Sirt-1, Sirt-3, and Sirt-5 protect the cell from ROS by acting on nuclear factor E2-related factor 2 (NRF2) which regulates the expression of several antioxidant and detoxification genes^53^. Whereas ROS were upregulated, downregulation of NO generation and iNOS expression with increasing patient age was demonstrated in the present study. NO bioactivity has been discussed as a determinant of human longevity and to protect against age-related degeneration^29^. Among others, this was based on the observation that the decrease in NO bioavailability with aging is especially apparent in sedentary individuals, whereas older, physically active individuals, maintain higher levels of NO with advancing age^11^. Recently it has been shown that non-thermal atmospheric pressure plasma (NTAPP) supported ASC stemness, the capability to differentiate into adipocytes and avoided cellular senescence by activating NO response pathways^54^. Moreover, electromagnetic stimulation of bone marrow-derived mesenchymal stem cells stimulated cell proliferation via transient NO production and extracellular signal regulated kinase 1/2 activation, which was more apparent in stem cells isolated from older patients than from young patients^55^. In contrast, previous studies showed that upon replicative senescence critical components of the iNOS mediated NO synthase pathway were upregulated in hASCs^56^, suggesting mechanistic differences between the healthy biological aging process and replicative senescence and potential dose-effects of produced oxidants.

In the present study chemical induction of adipogenesis resulted in gene activation of adipogenic genes and Oil Red O-positive adipocytes. Despite expression of stem cell markers also in the cohort of elderly patients, a decline in adipogenic capacity with increasing age was observed. This is in consent with a previous study of^5^ on human ASCs and rabbits^57^, but contrasts the studies of^34^ and^58^, who reported that the adipogenic differentiation potential seemed to remain on the same level throughout the whole ageing process. Notably the capacity of adipogenic differentiation is related to lamin A levels since overexpression of both wild-type and mutant lamin A inhibited lipid accumulation, triglyceride synthesis and expression of adipogenic markers^59^. Recently it was shown, that progeria caused by Werner syndrome was associated with inhibition of adipogenesis in subcutaneous fat tissue^60^. It is well known that during aging visceral adipose tissue increases, whereas a significant decrease in peripheral subcutaneous adipose tissue occurs. The decay in subcutaneous adipose tissue in old-aged individuals may be due to an aging-dependent regulatory cell (ARC) population secreting pro-inflammatory chemokines to inhibit proliferation and differentiation of neighboring adipose precursors as previously evidenced^61^. A previous study reported that primary human senescent fat progenitors secrete activin A and directly inhibit adipogenesis in non-senescent progenitors^62^. Recently it was demonstrated that knockdown of Sirt-1 during adipogenic differentiation resulted in impaired oxidative stress response. Increased oxidative stress with H_2_O_2_ or SOD2 knockdown phenocopied Sirt-1 inhibition^19^. Moreover, studies on mouse embryonic stem cells demonstrated that knockdown of Sirt-1 and incubation with the Sirt-1 inhibitor EX-527 inhibited adipogenic differentiation, which is in line with the data of the present study showing an age-dependent decline of Sirt-1 expression and impaired capacity of adipogenesis. In endothelial cells differentiated from induced pluripotent stem cells (iPSCs) overexpression of Sirt-1 function preserved proliferative capacity through overcoming early cell senescence by increasing NO generation^63^.

In the present study, adipogenic induction of ASCs resulted in upregulation of Sirt-1 and Sirt-3 protein expression, which was not affected by the Sirt-1 activator YK-3-237 or the Sirt-1 inhibitor EX-527. YK-3-237 has been shown to deacetylate mutant p53 (mtp53) through Sirt-1^64^, whereas EX-527 binds after the dislocation of nicotinamide from Sirt-1 and prevents the release of deacetylated peptide and *O*-acetyl-ADP-ribose, the products of enzyme-catalyzed deacetylation^65^. Therefore, the data of the present study suggest that the deacetylase function of Sirt-1 is not important for adipogenesis-mediated upregulation of Sirt-1 and Sirt-3. Moreover, activation or inhibition of Sirt-1-mediated acetylation was not affecting mRNA expression of *LPL* and mRNA as well as protein expression of PPAR-γ, which are crucial components of the adipogenic differentiation process. Sirt-1 has been reported to repress *PPAR-γ* by docking with its cofactors NCoR (nuclear receptor co-repressor) and SMRT (silencing mediator of retinoid and thyroid hormone receptors)^66^.

A further key player in the adipogenic process is adiponectin protein, synthesized and secreted predominantly by adipocytes into the peripheral blood. Several studies reported that circulating adiponectin levels are inversely related with body weight, especially visceral fat accumulation, and low circulating adiponectin levels are associated with obesity-related diseases^67^. Sirt-1 increases adiponectin transcription in adipocytes by activating Foxo1 and enhancing Foxo1 and C/EBPα interaction^68^. In mouse bone marrow stromal cells Sirt-3 overexpression stimulated adipogenesis and adiponectin expression, whereas inhibition was observed by the Sirt-3 inhibitor 3-TYP^69^. In the present study adipogenic differentiation resulted in upregulation of adiponectin expression with maximum expression in the middle-aged group of patients and a significant decrease in old-aged patients which may be associated with the observed decay of Sirt-1 expression in elderly patients. Undifferentiated ASCs were devoid of adiponectin expression (data not shown). Notably treatment with the Sirt-1,2 antagonist Sirtinol significantly downregulated adiponectin protein, whereas upreguation was observed by the Sirt-1 activator YK-3-237 expression. Consequently, Sirt-1 activation stimulated and Sirt-1 inhibition inhibited the differentiation of Oil Red O-positive adipocytes. This observation supports previous research showing that adiponectin is an autocrine factor in adipose tissues which promotes cell proliferation and differentiation from preadipocytes into adipocytes and augments programmed gene expression responsible for adipogenesis^30^.

## Conclusions

ASCs derived from patients at an advanced age show reduced proliferation, increased oxidative stress, downregulation of Sirt-1 and decreased capacity of adipogenic differentiation. This is accomplished by Sirt-1-mediated inhibition of adiponectin expression.

### Supplementary Information

Supplementary Figure S1.

Supplementary Figures.

Supplementary Table S1.

## Data Availability

The data that support the findings of this study are not openly available due to reasons of sensitivity and are available from the corresponding author upon reasonable request. Data are located in controlled access data storage at Friedrich Schiller University Jena.
