# Supplementary figures and images for: Oxidative stress and regulation of adipogenic differentiation capacity by sirtuins in adipose stem cells derived from female patients of advancing age
Source: Sci Rep. 2024 Aug 27;14:19885. doi: 10.1038/s41598-024-70382-x (PMC11349916; doi:10.1038/s41598-024-70382-x)

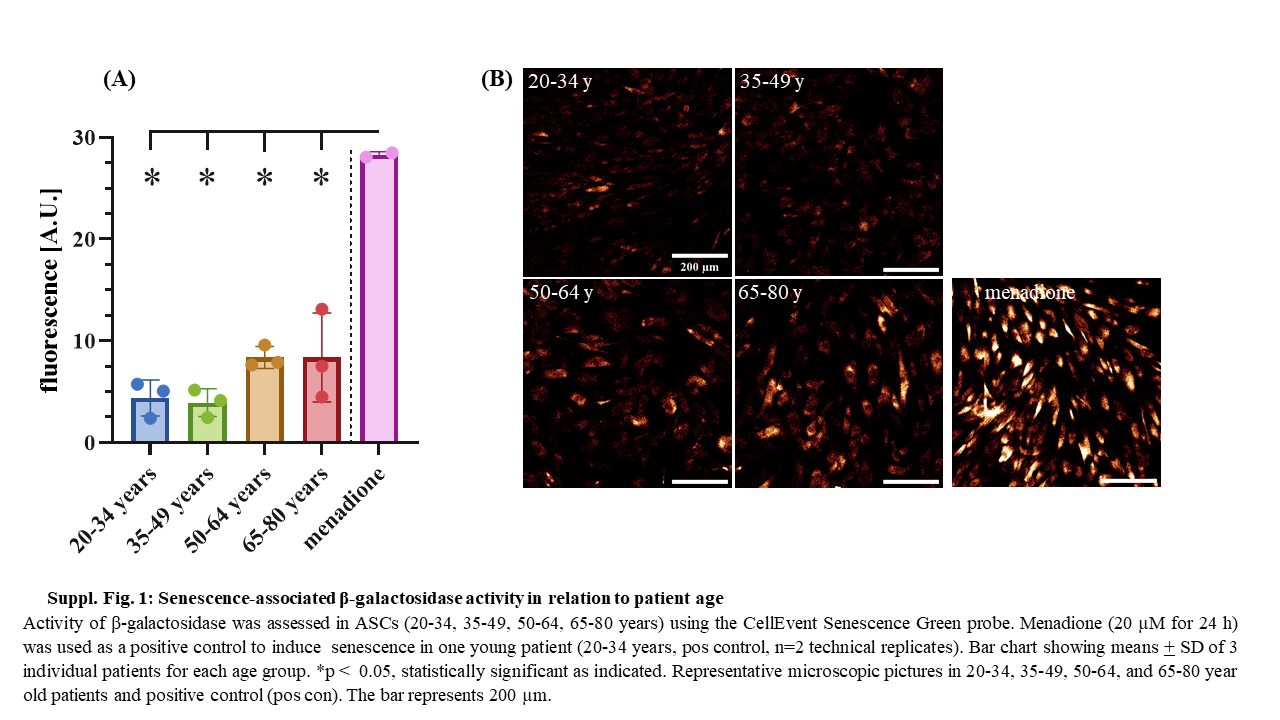

Supplement: Supplementary file 1 — Supplementary Figure S1. [file 41598_2024_70382_MOESM1_ESM.tif]

**Fig. 2A**

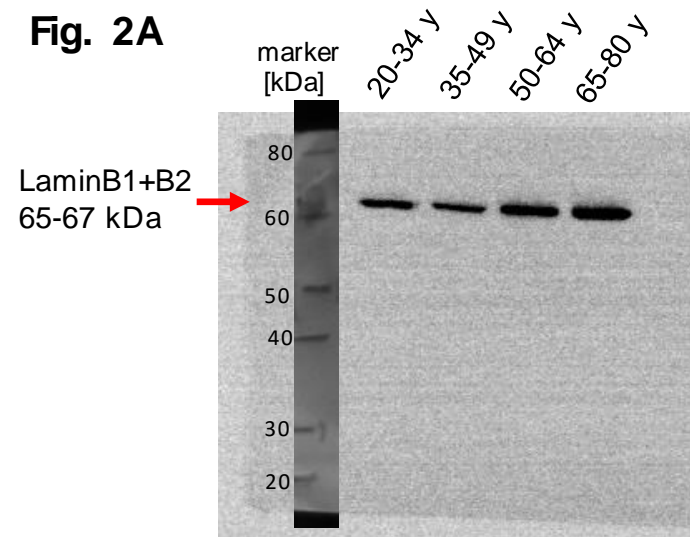

**Fig. 2B**

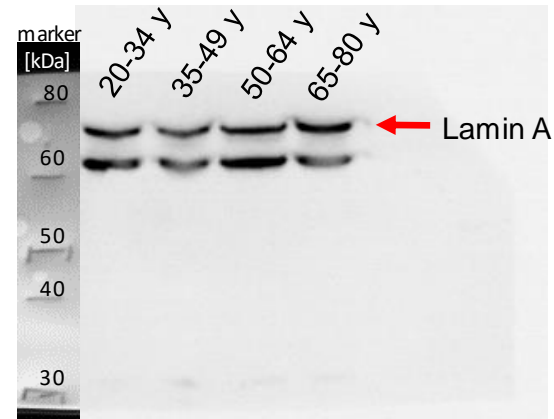

**Fig. 2C**

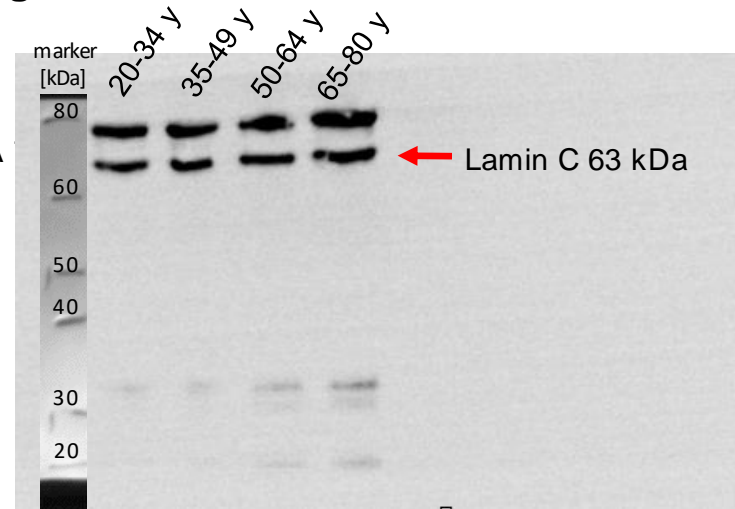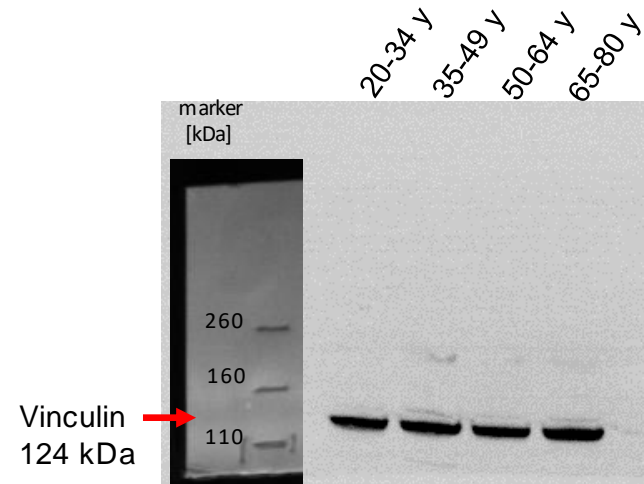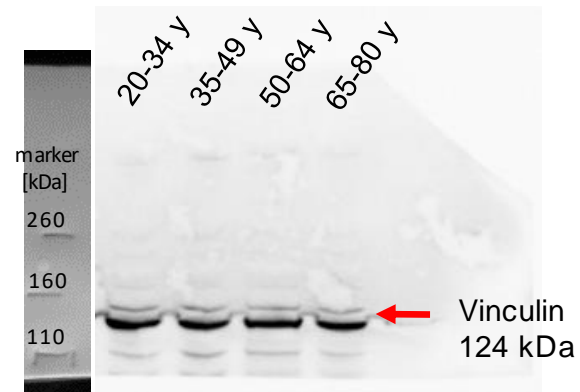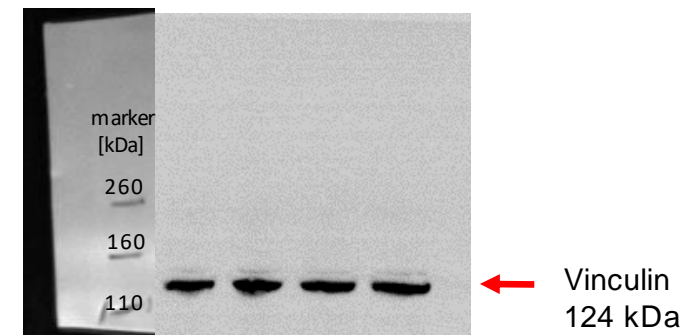

**Fig. 2D**

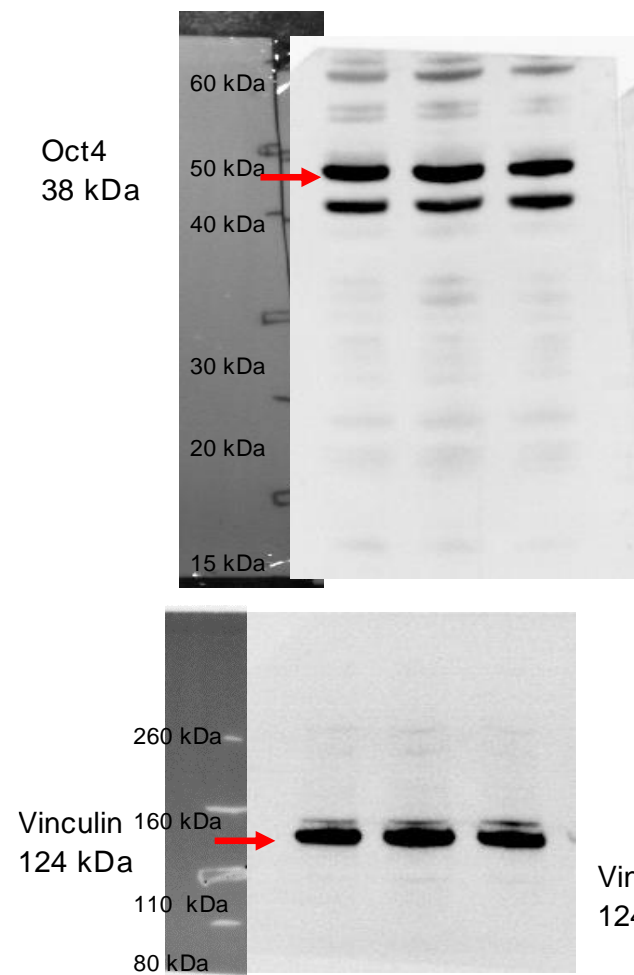

**Fig. 2E**

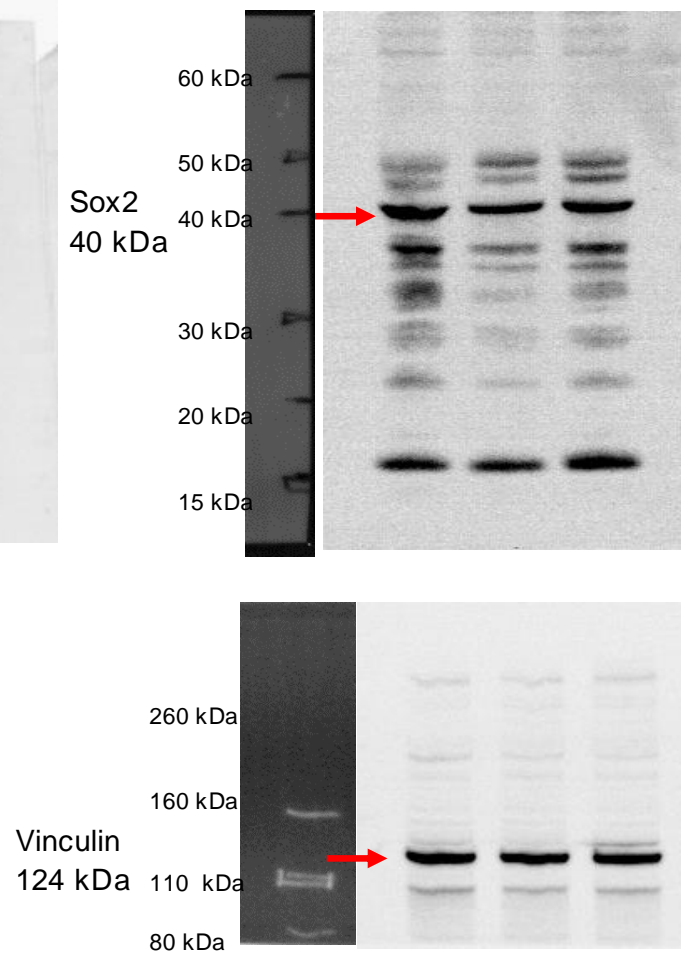

**Fig. 2F**

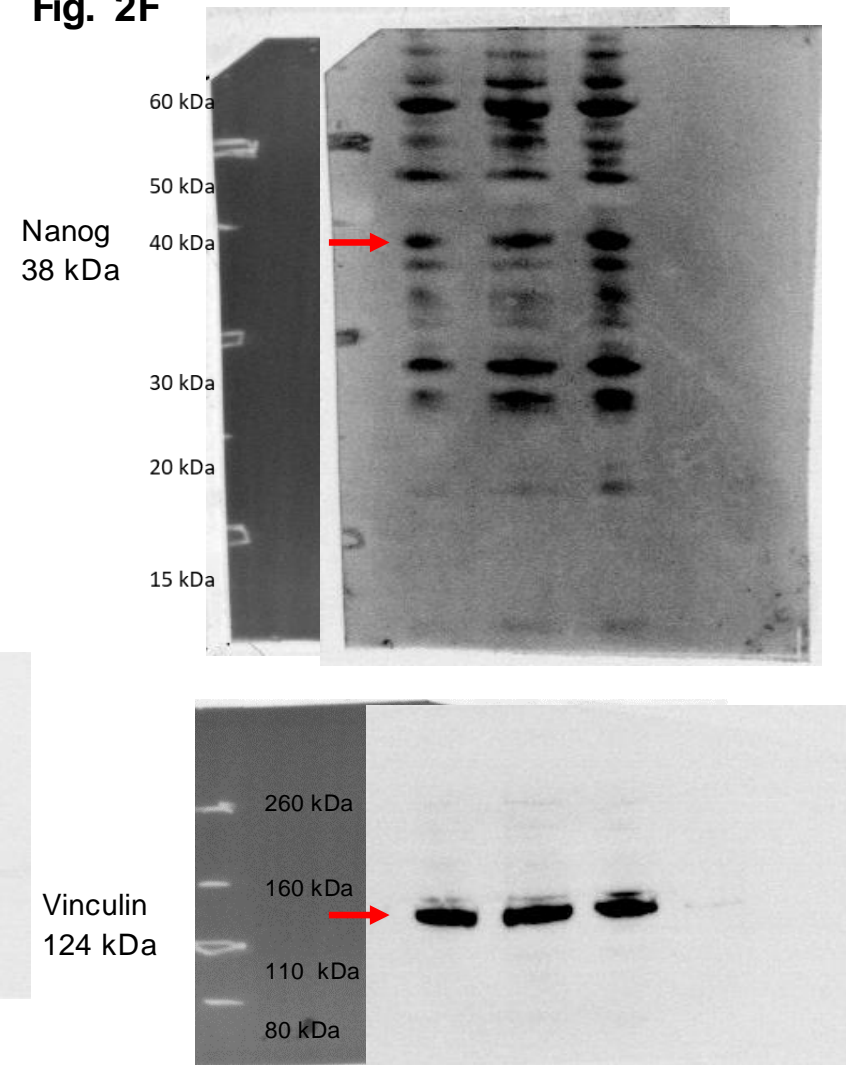

**Fig. 3A**

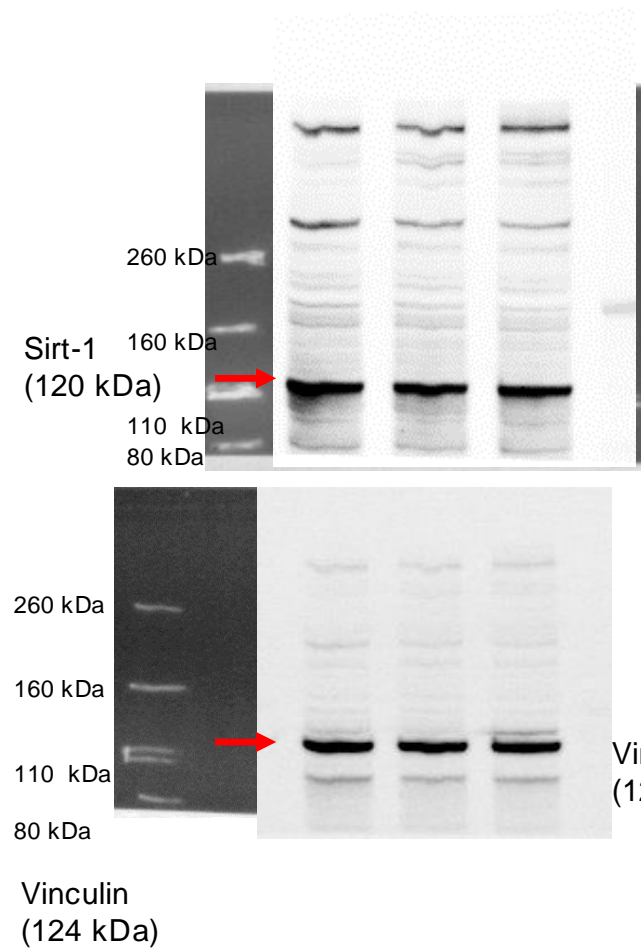

**Fig. 3B**

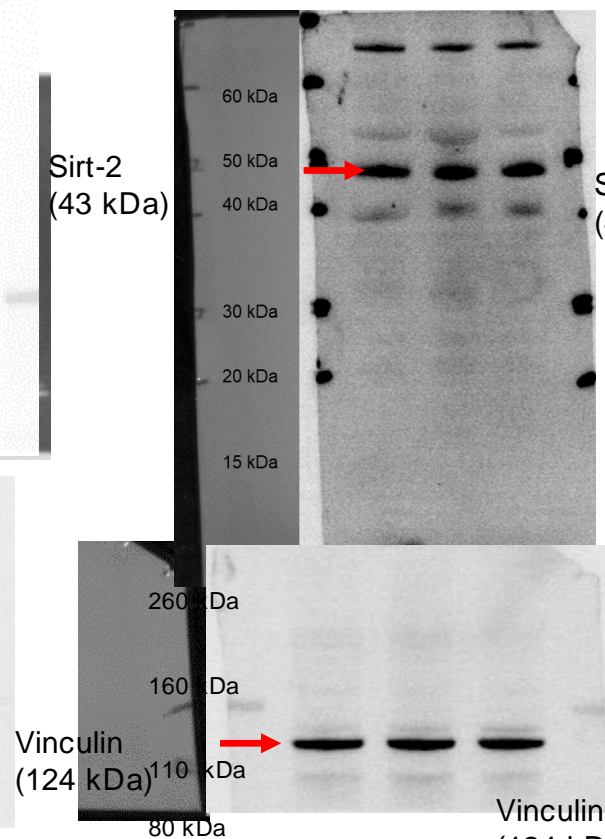

**Fig. 3C**

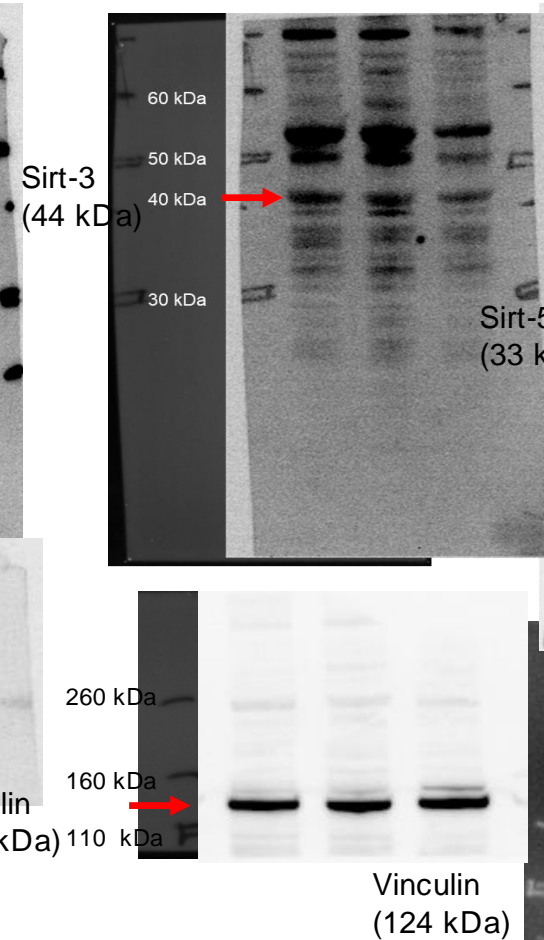

**Fig. 3D**

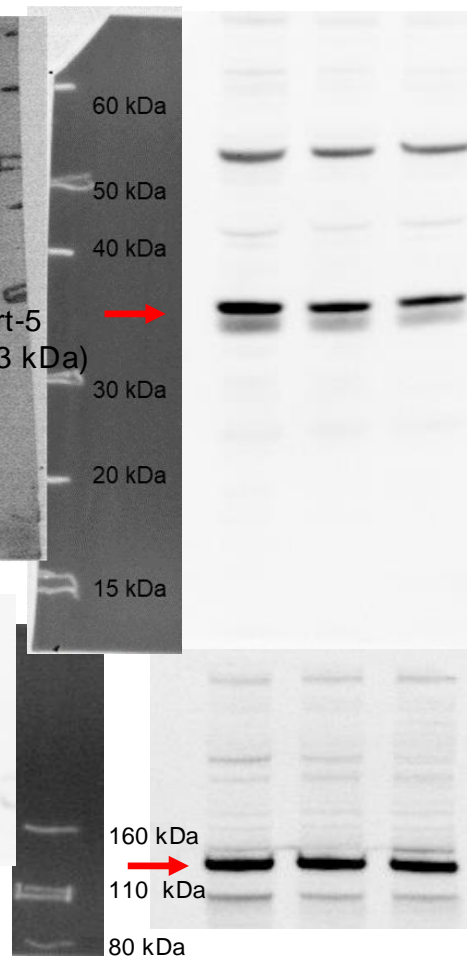

**Fig. 4C-F**

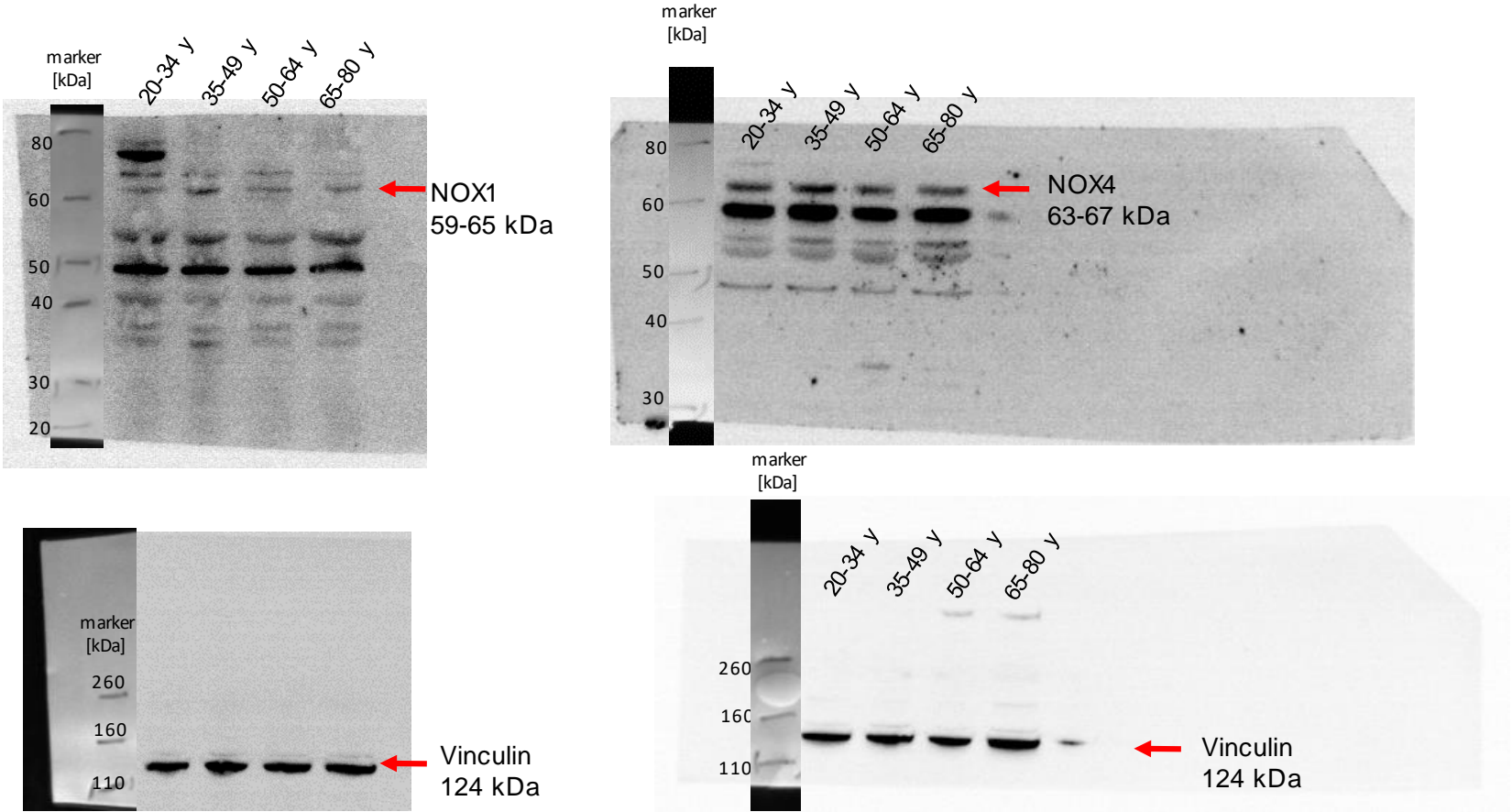

**Fig. 4C-F** marker  
[kDa]

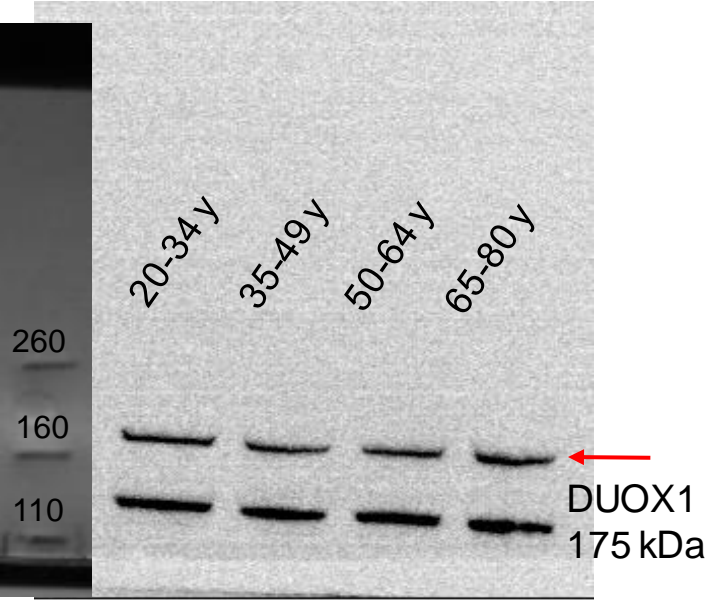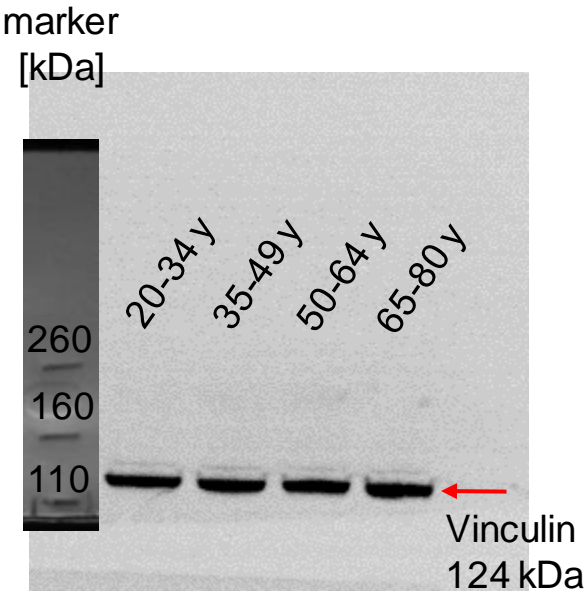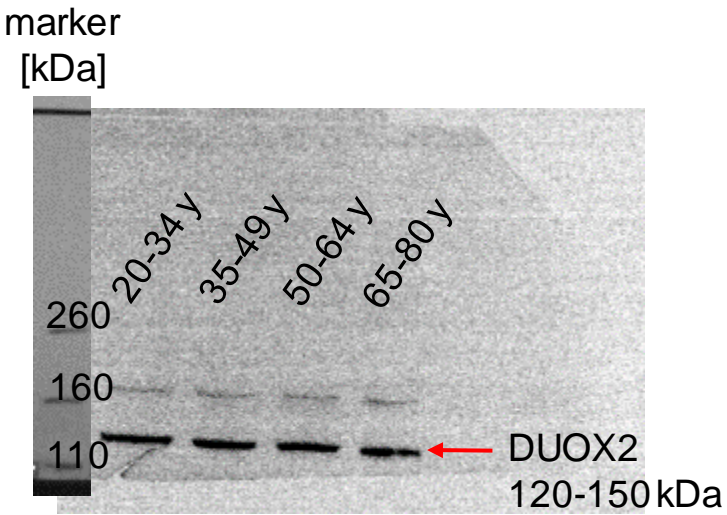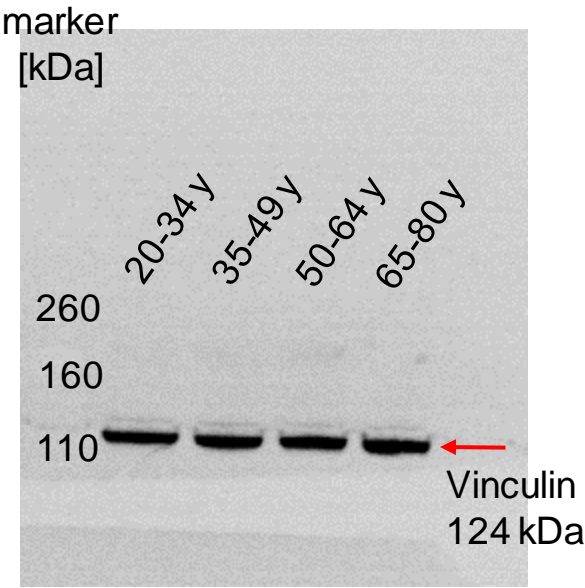

Fig. 4H

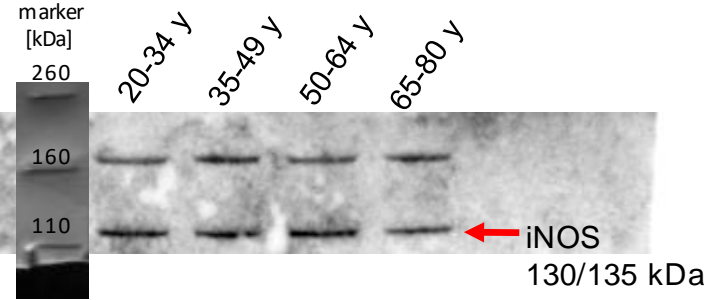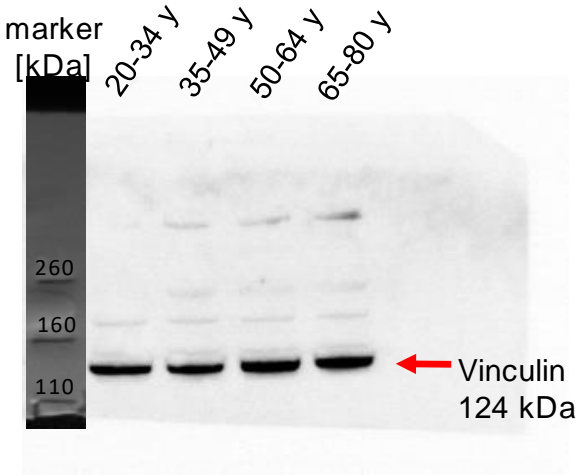

**Fig. 5I**

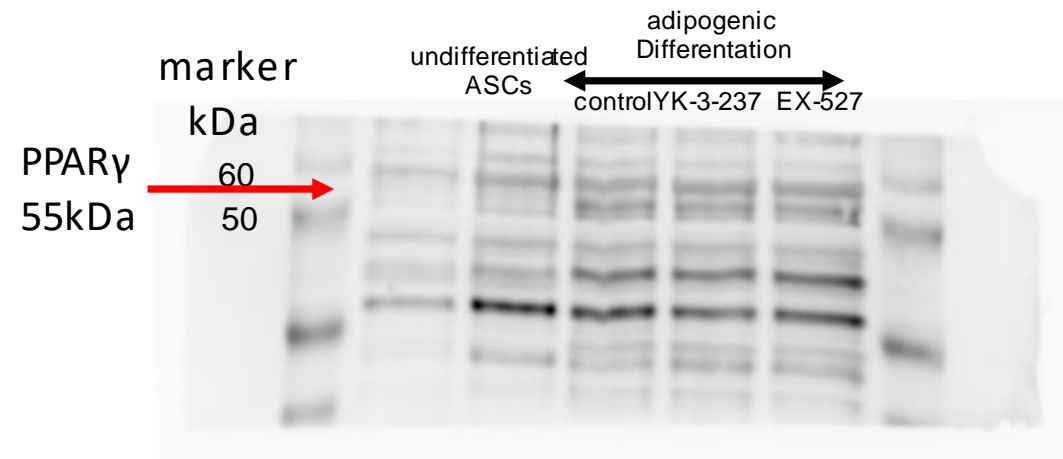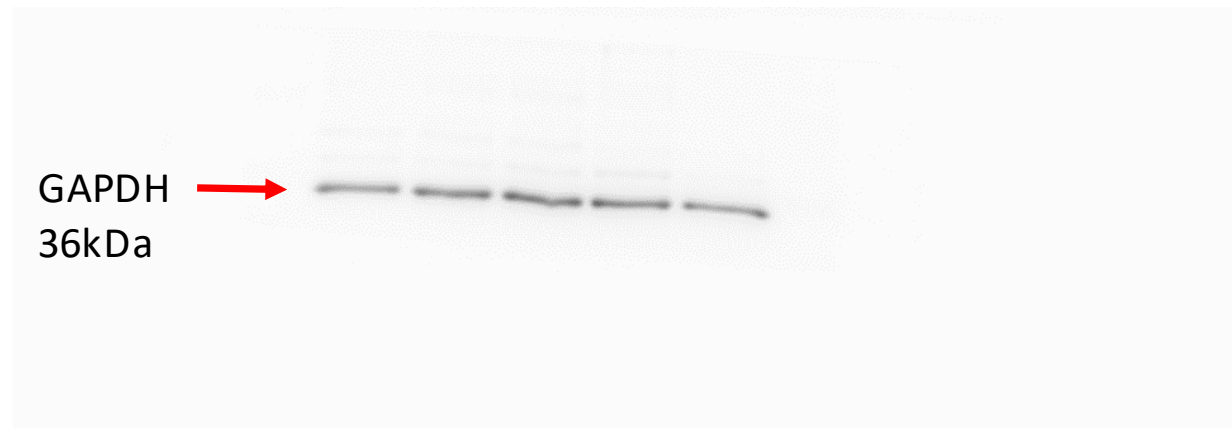

**Fig. 5J**

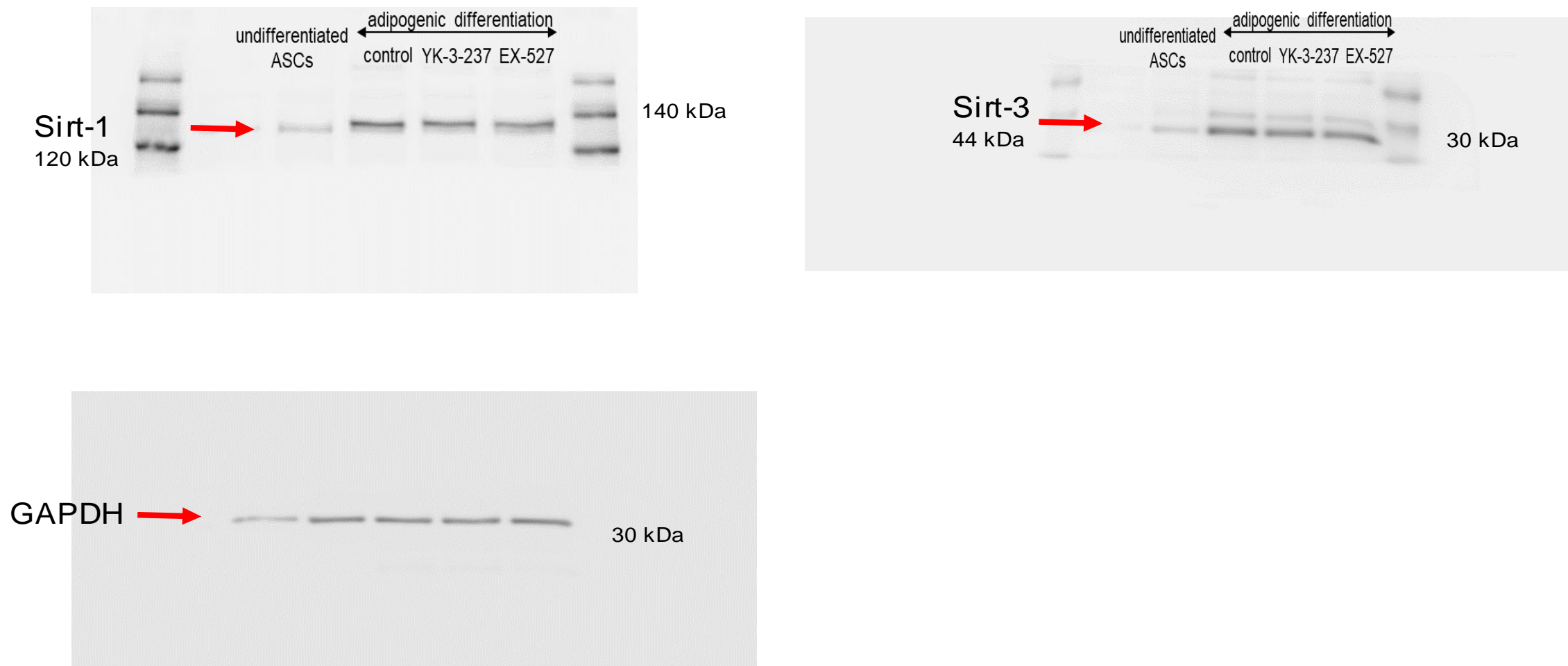

Fig. 6A

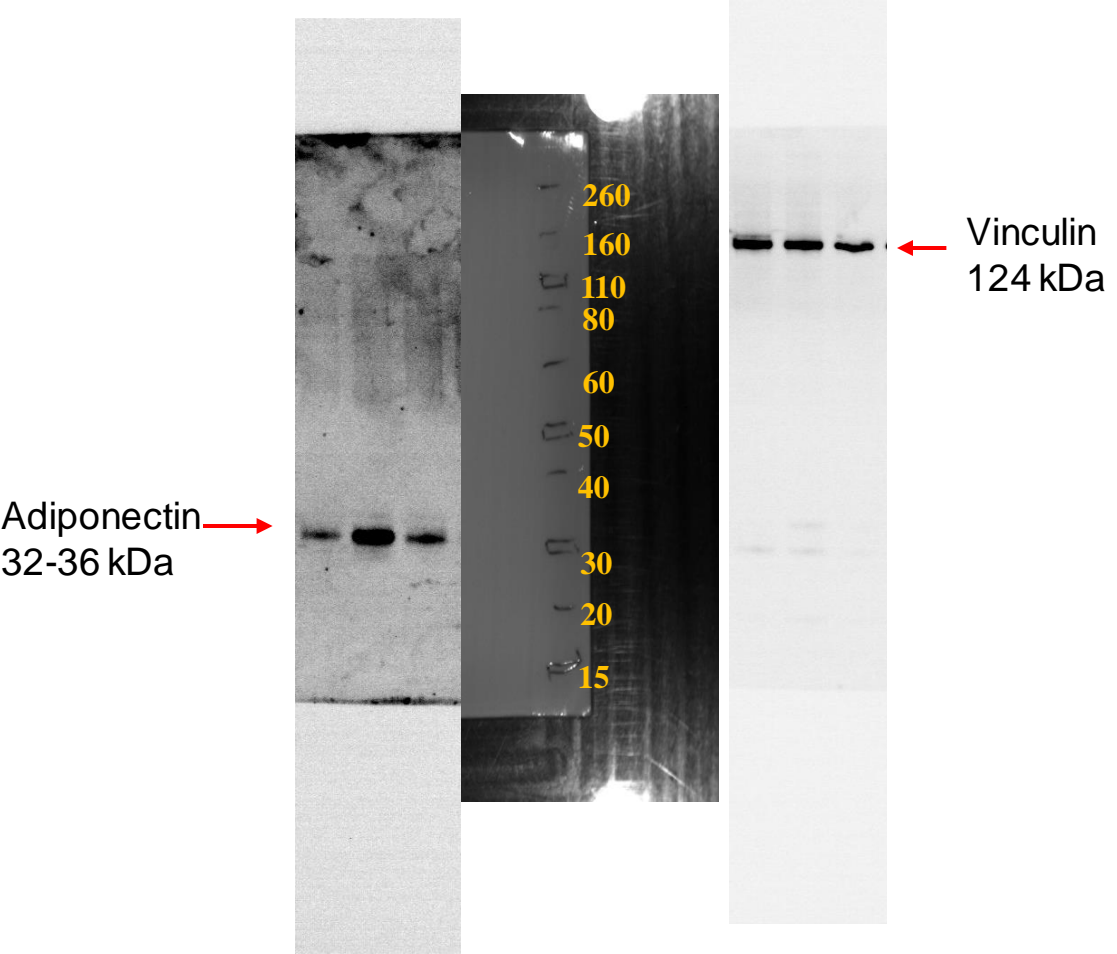

Fig. 6B

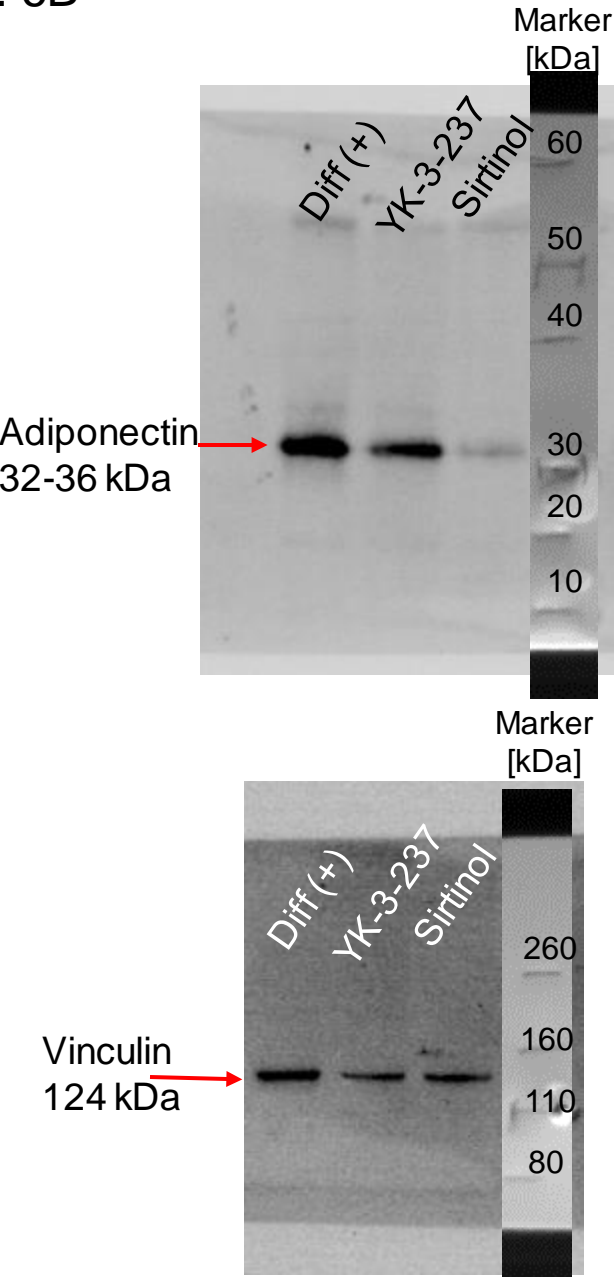

Supplement: Supplementary file 2 — Supplementary Figures. [file 41598_2024_70382_MOESM2_ESM.pdf]
