# Supplementary material for: Oxidative stress and regulation of adipogenic differentiation capacity by sirtuins in adipose stem cells derived from female patients of advancing age
Source: Sci Rep. 2024 Aug 27;14:19885. doi: 10.1038/s41598-024-70382-x (PMC11349916; doi:10.1038/s41598-024-70382-x)
Supplement: Supplementary file 3 — Supplementary Table S1. [file 41598_2024_70382_MOESM3_ESM.docx]

| Code no. | Age at surgery | Gender | Site of surgery | Tumor | Diabetes type 2 | Smoker |
| --- | --- | --- | --- | --- | --- | --- |
| hADSC15 | 24 | female | thigh | no | no | no |
| hADSC16 | 26 | female | thigh | no | no | no |
| ASC05 | 27 | female | thigh | no | no | no |
| hASC24 | 30 | female | abdomen | no | no | yes |
| hADSC36 | 30 | female | abdomen | no | no | no |
| hADSC39 | 31 | female | thigh | no | no | yes |
| hADSC04 | 31 | female | hip | no | no | no |
| hADSC31 | 36 | female | thigh | no | no | yes |
| hASC27 | 37 | female | thigh | no | no | no |
| hADSC23 | 38 | female | thigh | no | no | yes |
| hADSC18 | 38 | female | thigh | no | no | no |
| hADSC46 | 38 | female | thigh | no | no | no |
| hADSC28 | 40 | female | thigh | no | no | yes |
| hADSC35 | 40 | female | lower leg | no | no | no |
| hADSC45 | 40 | female | lower leg | no | no | no |
| hADSC19 | 53 | female | abdomen | no | no | no |
| hASC29 | 56 | female | thigh | no | no | no |
| hADSC13 | 57 | female | thigh | no | no | no |
| hADSC14 | 57 | female | abdomen | no | no | no |
| hADSC20 | 57 | female | n/a | no | no | no |
| ASC41 | 58 | female | thigh | no | no | no |
| ASC40 | 59 | female | thigh | no | no | no |
| hADSC27 | 60 | female | thigh | no | no | no |
| hADSC43 | 61 | female | lower leg | no | no | no |
| hADSC32 | 67 | female | thigh | no | no | no |
| hADSC XIXn | 69 | female | n/a | yes | no | yes |
| ADSC XXII | 70 |  | upper arm | yes | no | no |
| hADSC34 | 71 | female | abdomen | no | no | no |
| hADSC IXn | 70 | female | neck | yes | no | yes |
| ADSC X | 75 | female | right scapula | yes (squamous cell carcinoma) | n/a | n/a |
| hADSC VIIIn | 76 | female | thigh | yes | no | no |
